# *GhWRKY68* Reduces Resistance to Salt and Drought in Transgenic *Nicotiana benthamiana*

**DOI:** 10.1371/journal.pone.0120646

**Published:** 2015-03-20

**Authors:** Haihong Jia, Chen Wang, Fang Wang, Shuchang Liu, Guilin Li, Xingqi Guo

**Affiliations:** State Key Laboratory of Crop Biology, College of Life Sciences, Shandong Agricultural University, Tai’an, Shandong, PR China; Key Laboratory of Horticultural Plant Biology (MOE), CHINA

## Abstract

The WRKY transcription factors modulate numerous physiological processes, including plant growth, development and responses to various environmental stresses. Currently, our understanding of the functions of the majority of the WRKY family members and their possible roles in signalling crosstalk is limited. In particular, very few WRKYs have been identified and characterised from an economically important crop, cotton. In this study, we characterised a novel group IIc WRKY gene, *GhWRKY68*, which is induced by different abiotic stresses and multiple defence-related signalling molecules. The *β*-glucuronidase activity driven by the *GhWRKY68* promoter was enhanced after exposure to drought, salt, abscisic acid (ABA) and H_2_O_2_. The overexpression of *GhWRKY68* in *Nicotiana benthamiana* reduced resistance to drought and salt and affected several physiological indices. *GhWRKY68* may mediate salt and drought responses by modulating ABA content and enhancing the transcript levels of ABA-responsive genes. *GhWRKY68*-overexpressing plants exhibited reduced tolerance to oxidative stress after drought and salt stress treatments, which correlated with the accumulation of reactive oxygen species (ROS), reduced enzyme activities, elevated malondialdehyde (MDA) content and altered ROS-related gene expression. These results indicate that *GhWRKY68* is a transcription factor that responds to drought and salt stresses by regulating ABA signalling and modulating cellular ROS.

## Introduction

In the natural environment, plants often simultaneously confront a great variety of abiotic and biotic stresses. To cope with these stresses, they have evolved sophisticated defence mechanisms. Transcriptional modulation is vital for the complex genetic and biochemical networks to respond to stress. A number of transcription factors (TFs) have been shown to participate in regulating defence responses [1, 2].

WRKY TFs are the most important TFs in plants, and they contain one or two highly conserved WRKYGQK sequences at the N-terminus and a zinc finger motif at the C-terminus [3]. Based on the number of WRKY domains and the primary structure of the zinc-finger motif, WRKY members can be subdivided into three major groups (I–III), and group II can be further split into five subgroups (IIa-e) [4]. It is generally assumed that WRKY TFs act as major regulatory proteins by specifically binding to the W-box [TTGAC(C/T)] to regulate gene expression [2, 3, 5].

There is an increasing amount of evidence that WRKY TFs are key regulators in the complex signalling and transcriptional networks of plant defences [6–9]. Previous studies have examined the roles of plant WRKY proteins in response to biotic stress. For example, *BnWRKY33* plays an important role in *B*. *napus* defence against *S*. *sclerotiorum*, which is associated with the activation of the salicylic acid (SA)- and jasmonic acid (JA)-mediated defence responses [10]. *CaWRKY40* is regulated by SA, JA and ethylene (ET) signalling, and it plays an important role in the regulation of tolerance to heat stress and resistance to *Ralstonia solanacearum* infection [11]. Over the last several years, an increased amount of evidence has shown that WRKY proteins are also involved in modulating abiotic stress tolerance [12–15]. In *Arabidopsis*, *WRKY25*, *WRKY26* and *WRKY33* play important roles in the response to heat stress [16]. Constitutive expression of *WRKY57* can improve drought tolerance [14]. Transgenic *Arabidopsis* overexpressing *TaWRKY2* or *TaWRKY19* displayed improved salt and drought tolerance [17]. Three soybean WRKY-type transcription factor genes conferred differential tolerance to abiotic stresses in transgenic *Arabidopsis* plants. For example, *GmWRKY21* transgenic plants were tolerant to cold stress, whereas *GmWRKY54* conferred salt and drought tolerance. Transgenic plants overexpressing *GmWRKY13* exhibited sensitivity to salt and mannitol stress [18]. ABO3/WRKY63 mediates responses to abscisic acid (ABA) and drought tolerance in *Arabidopsis* [19]. The alleles OsWRKY45-1 and OsWRKY45-2 play different roles in ABA signalling and cold, salt and drought stresses adaptation in rice [20]. Recently, Yan reported that GhWRKY17 responds to drought and salt stress through ABA signalling and the control of cellular ROS production in cotton [21]. Although evidence that WRKY proteins are involved in abiotic stress is increasing, the understanding of the roles of these proteins in the responses to abiotic stress is progressing relatively slowly [17], and the challenge to elucidate the molecular mechanisms of defence remains. Moreover, there is little information about *WRKY* genes in non-model plants.

Abscisic acid (ABA), an important phytohormone, is a key signal for regulating a range of plant physiological processes in response to various biotic and abiotic stresses. Osmotic stresses, including drought and high salinity, can trigger the ABA-dependent signalling pathway and ABA accumulation [22, 23]. An increased level of ABA activates downstream transcription factors and modulates the expression of various ABA-responsive genes [24, 25]. ABA also promotes cellular reactive oxygen species (ROS) production in *Arabidopsis* guard cells [26], and Zhang [27] reported that ROS positively regulates the ABA inhibition of stomatal opening. Extensive research has demonstrated that ROS are important signal transduction molecules, modulating plant development, growth, hormonal signalling, and defence [28, 29]. High ROS concentrations contribute to ROS-associated injury [30], and the regulation of ROS levels is crucial for abiotic stress tolerance in plants [31]. It is important to examine the roles of WRKY TFs in cross-network signalling between ROS and ABA in the response to drought and salt stress.

Cotton is an important source of natural fibre used in the textile industry, and it is particularly susceptible to waterlogging stress [32]. *Gossypium hirsutum L*. represents more than 95% of the cotton cultivated worldwide [33]. In cotton, only a small number of WRKY TFs have been isolated and characterised. Therefore, understanding the underlying roles of WRKY proteins in the tolerance of osmotic stress is an important global problem for breeding programs. In the present study, a group IIc WRKY gene, *GhWRKY68*, was isolated from cotton (*G*. *hirsutum L*.). The gene can be induced by abiotic stresses and multiple defence-related signalling molecules. Many abiotic stress-responsive elements were observed in the promoter region of this gene, and *β*-glucuronidase activity driven by the promoter was enhanced by treatments with drought, salt, ABA and H_2_O_2_. The overexpression of *GhWRKY68* in *Nicotiana benthamiana* significantly reduced resistance to drought and salt modulated by ABA signalling and the regulation of ROS. Hence, this study was conducted with the aim of understanding the mechanism by which the WRKY TFs regulate plant responses to drought and salt stress.

## Results

### Identification and sequence analysis of *GhWRKY68*

**Fig 1:**
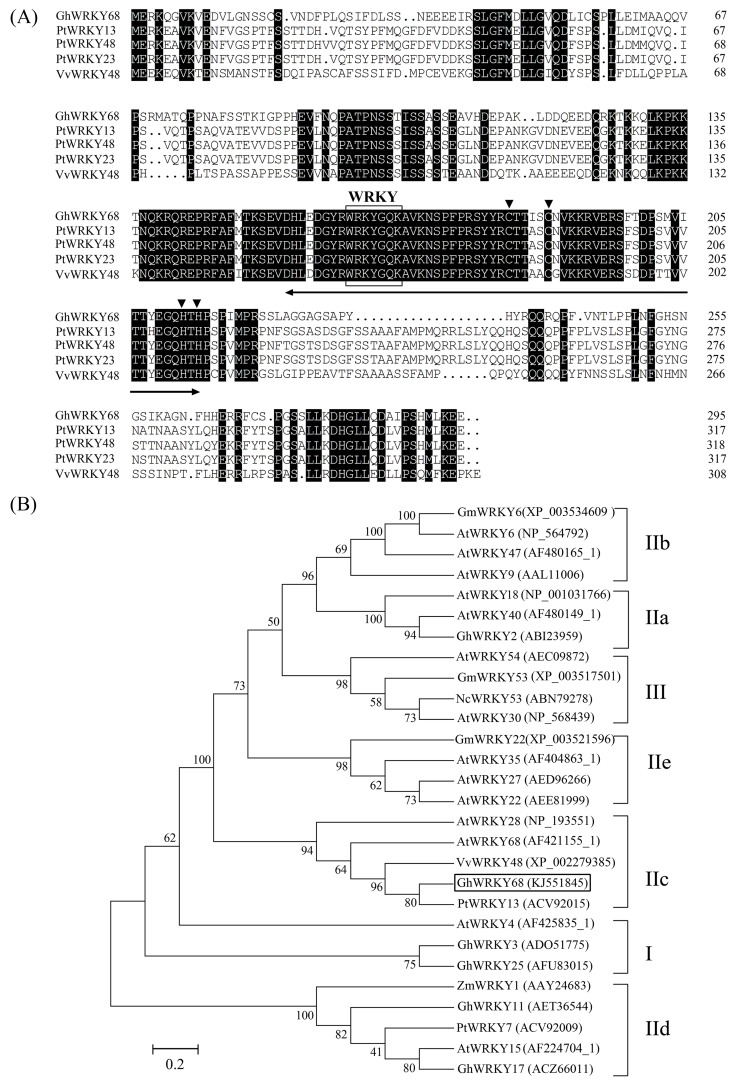
Sequence and phylogenetic analyses of GhWRKY68. (A) Sequence alignment of the deduced GhWRKY68 protein with *PtWRKY48*, *PtWRKY23*, *VvWRKY48*, and *PtWRKY13*. Identical amino acids are shaded black. The approximately 60-amino acid WRKY domain is marked by the two-headed arrow. The C and H residues in the zinc-finger motif (C-X4-5-C-X22-23-H-X1-H) are marked by triangles. The highly conserved amino acid sequence WRKYGQK in the WRKY domain is boxed. (B) Phylogenetic analysis of WRKY proteins from different species. The amino acid sequences were subjected to Clustal W using the neighbour-joining method in MEGA 4.1. GhWRKY68 is highlighted in the box. The gene name is followed by the protein ID. The species of origin of the WRKYs are indicated by the abbreviations before the gene names: At *Arabidopsis thaliana*, Gh *Gossypium hirsutum*, Pt *Populus tomentosa*, Vv *Vitis vinifera*, Zm *Zea mays*, Gm *Glycine max* and Nc *Noccaea caerulescens*.

Based on the conserved region of plant stress-related WRKY genes, a pair of degenerate primers, WP1 and WP2, was designed, and a putative WRKY fragment was isolated. Next, the rapid amplification of cDNA ends by PCR (RACE-PCR) was used to amplify the 5′ untranslated region (UTR) and the 3′ UTR. Finally, the deduced full-length cDNA sequence consisting of 1118 bp was retrieved. Because *AtWRKY68* is the *Arabidopsis* WRKY gene most closely related to this putative cotton WRKY gene, we designated this new WRKY gene as *GhWRKY68* (GenBank, KJ551845). The gene encoded a protein with a predicted relative molecular mass of 33.186 kDa and a theoretical isoelectric point of 8.71. Multi-alignment analysis revealed that the deduced WRKY protein was closely related to other plant WRKY proteins, sharing 57.14, 57.01, 51.90 and 57.32% homology with PtWRKY48 (XP_002301524), PtWRKY23 (ABK41486.1), VvWRKY48 (XP_002279385), and PtWRKY13 (ACV92015), respectively. Similar to the other WRKY TFs, GhWRKY68 has one WRKY domain that contains the highly conserved amino acid sequence WRKYGQK and a single putative zinc finger motif (C-X_4–5_-C-X_22–23_-H-X_1_-H) (Fig. 1A). Phylogenetic analysis further revealed the evolutionary relationship to other WRKYs from various plant species, suggesting that GhWRKY68 belongs to Group IIc of the WRKY family (Fig. 1B). In addition, with a pair of specific primers, WQC1 and WQC2, the *GhWRKY68* genomic sequence (KJ551846) was amplified. The sequence consisted of 2543 bp interrupted by two introns of 228 and 138 bp. Currently, there is no other study on *GhWRKY68* from cotton; therefore, we characterised this gene.

### Characterisation of GhWRKY68 as a transcription factor

**Fig 2:**
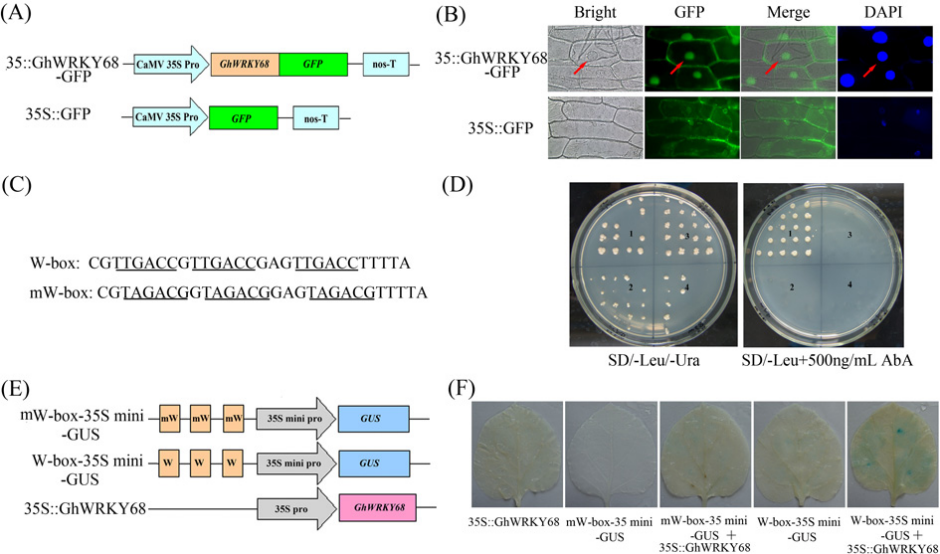
Characterisation of GhWRKY68 as a transcription factor. (A) Schematic diagram of the 35S-GhWRKY68::GFP fusion protein construct and the control 35S-GFP construct. GFP was fused in frame to the C terminus of GhWRKY68. (B) Transient expression of the 35S-GhWRKY68::GFP and 35S-GFP constructs in onion epidermal cells 24 h after particle bombardment. Green fluorescence was observed using a fluorescence microscope, and the nuclei of the onion cells were visualised by DAPI staining. (C) Sequence of the triple tandem repeats of the W-box and mW-box. (D) Transactivation analysis of GhWRKY68 with the yeast one-hybrid assay using the 3×W-box or mW-box as bait. Yeast cells carrying pGAD-GhWRKY68 or pGAD7 were grown on SD/-Leu/-Ura or SD/-Leu containing 500 ng/ml AbA. 1, pAbAi-W-box/pGAD-GhWRKY68, 2, pAbAi-W-box/pGAD7, 3, pAbAi-mW-box/pGAD-GhWRKY68, and 4 pAbAi-mW-box/pGAD7. (E) Schematic diagram of the reporter and effector constructs used for co-transfection. (F) Histochemical analysis of co-transfected *N*. *benthamiana* leaves. Fully expanded leaves from 8-week-old *N*. *benthamiana* were *agro*-infiltrated with the indicated reporter and effector at an OD_600_ of 0.6. GUS staining was performed 3 days after the transformation.

Using the Nuc-PLoc program and the CELLO version 2 program, we predicted that GhWRKY68 was localised in the nucleus. To test this prediction, 35S::GhWRKY68-GFP and 35S::GFP plasmids were constructed, and the latter was used as the control (Fig. 2A). Then, the 35S::GhWRKY68-GFP and 35S::GFP plasmids were introduced into onion epidermal cells. The fluorescence was observed by confocal microscopy. The nuclei were stained with DAPI. As shown in Fig. 2B, onion epidermal cells carrying the 35S::GhWRKY68-GFP plasmid emitted fluorescence only in the nuclei, whereas the 35S::GFP control exhibited GFP signals in both the cytoplasm and the nuclei. These results demonstrated that the GhWRKY68 protein was localised in the nucleus.

Numerous studies have demonstrated that WRKY TFs modulate protein expression by binding to the W-box [TTGAC(C/T)], which is present in the promoters of defence-associated genes as well as in many WRKY genes [3]. To test whether this binding also applies to *GhWRKY68*, a yeast one-hybrid experiment was performed. Three tandem repeats of the W-box (TTGACC) or the mW-box (TAGACG) (Fig. 2C) were inserted into the pAbAi vector and integrated into the genome of yeast strain Y1H gold with an Aureobasidin A resistance (AbA^r^) reporter gene (*AUR-1C*). A yeast effector vector, pGADT7-WRKY68, and an empty vector, pGADT7, were transformed into the yeast strain Y1H Gold carrying a pAbAi-W-box or a pAbAi-mW-box plasmid. All of the transformed yeast cells grew on leucine (Leu)- and uracile (Ura_-deficient synthetic dextrose (SD) medium (SD/-Leu/-Ura), confirming the success of the transformation (Fig. 2D). Only the yeast clones harbouring the pAbAi-W-box and pGAD-GhWRKY68 grew on SD/-Leu containing 500 ng/ml AbA (Fig. 2D). These results demonstrated that GhWRKY68 bound to the W-box element and functioned as a transcriptional activator in this yeast system.

To test whether *GhWRKY68* activates gene expression by interacting with the W-box in plant cells, we performed transient co-expression experiments. The reporter vector W-box-35S mini-GUS (Fig. 2E) was either transformed alone or with the effector plasmid 35S::GhWRKY68 (Fig. 2E) into *N*. *benthamiana* leaves using *Agrobacterium*-mediated transient expression followed by a GUS histochemical staining assay. The tobacco leaves co-transformed with W-box-35S mini-GUS and 35S::GhWRKY68 were stained dark blue. In contrast, leaves transformed with only the effector never stained blue, and leaves transformed with only the reporter vector showed a slight blue background (Fig. 2F). Thus, overexpression of *GhWRKY68* can activate the expression of GUS in *N*. *benthamiana* leaves in a W-box-dependent manner.

### *GhWRKY68* promoter analysis

To clarify the mechanism underlying the *GhWRKY68* expression patterns in response to multiple stresses, the 1118 bp promoter region of *GhWRKY68* (KJ551846) was isolated using inverse PCR (I-PCR) and nested PCR [34]. Many response elements for abiotic and biotic stress, tissue-specific expression, development and light were predicted by the database search programs PLACE and Plant-CARE (Table 1). Among these elements was MBS, a MYB binding site involved in drought tolerance in *Arabidopsis* [35]. An AREB cis-acting element was predominant in ABA-dependent gene expression [36]. These results suggest that *GhWRKY68* may play a role in the response to environmental stresses and in developmental pathways.

**Table 1:** Putative *cis*-acting elements of the promoter of *GhWRKY68*.

Cis*-element*	*Position*	*Sequence (5'-3')*	*function*
*Abiotic stress responsive element*			
ABRE	-90 (+)	CACGTG	cis-acting element involved in the abscisic acid responsiveness
HSE	-555 (+)	AAAAAATTTC	cis-acting element involved in heat stress responsiveness
TCA-element	-1056 (-)	CAGAAAAGGA	cis-acting element involved in salicylic acid responsiveness
GARE-motif	-150 (+)	AAACAGA	gibberellin-responsive element
LTR	-575 (+)	CCGAAA	cis-acting element involved in low-temperature responsiveness
MBS	-961(-)	CAACTG	MYB binding site involved in drought-inducibility
ARE	-733 (+)	TGGTTT	cis-acting regulatory element essential for the anaerobic induction
TC-rich repeats	-497 (-)	ATTTTCTTCA	cis-acting element involved in defense and stress responsiveness
*Pathogen/elicitor-related elements*			
Box-W1	-910 (+)	TTGACC	fungal elicitor responsive element
*Light responsive elements*			
GA-motif	-287 (-)-995 (-)	AAAGATGAAAGGAAGA	part of a light responsive element
GAG- motif	-942 (-)-991(-)	AGAGAGT	part of a light responsive element
GATA-motif	-378 (-)	AAGATAAGATT	part of a light responsive element
I-box	-378 (-)-380 (-)	GATAAGATAAAGATAAGA	part of a light responsive element
TCCC—motif	-950 (+)	TCTCCCT	part of a light responsive element
Sp1	-797(-)	GGGCGG	light responsive element
TCT-motif	-744(-)	TCTTAC	part of a light responsive element
*Tissue-specific and development-related elements*			
CCGTCC-box	-306(-)	CCGTCC	cis-acting regulatory element related to meristem specific activation
Skn-1_motif	-874(-)	GTCAT	cis-acting regulatory element required for endosperm expression
circadian	-279(+)	CAANNNNATC	cis-acting regulatory element involved in circadian control

**Fig 3:**
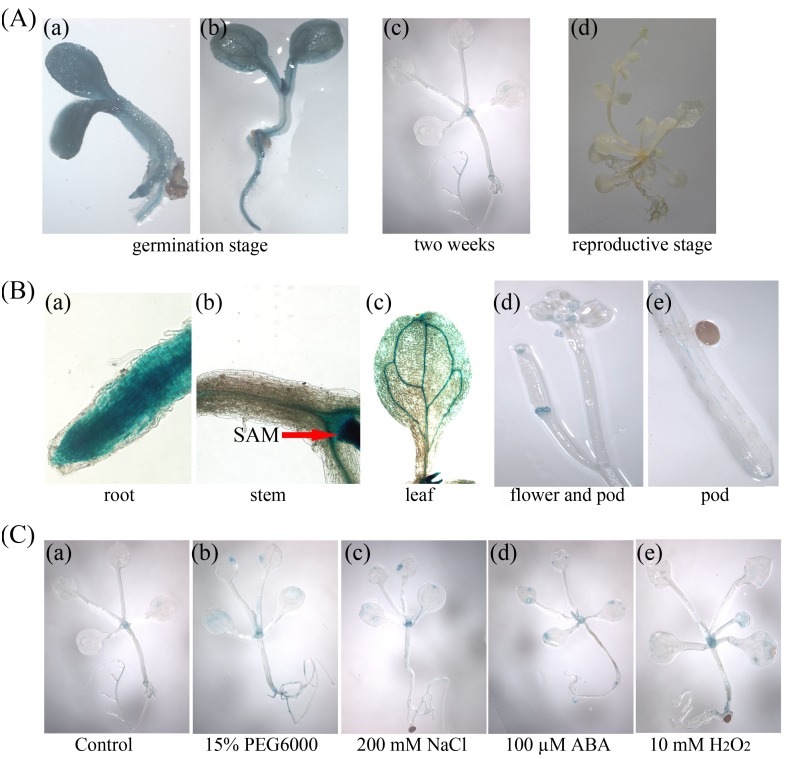
Histochemical analyses of GUS activity in ProGhWRKY68::GUS transgenic *Arabidopsis*. (A) ProGhWRKY68::GUS activity during different developmental stages. (B) Activity of the ProGhWRKY68::GUS construct in different tissue regions. (C) Histochemical assays of GUS activity in response to various stresses.

To test the activity of the *GhWRKY68* promoter, four independent transgenic *Arabidopsis* T_3_ lines harbouring the ProGhWRKY68::GUS construct were used for GUS histochemical staining assays. As shown in Fig. 3A, GUS staining was mainly detected in the germination stage, and weak GUS staining was observed at the reproductive stage. The tissue-specific regulation of the *GhWRKY68* promoter, assessed by GUS expression, was confined to the root, leaf and shoot apical meristem (SAM) of 2-week-old transgenic seedlings (Fig. 3B (a-c)) and to the flower and pod at the reproductive stage (Fig. 3B (d-e)). These results indicate that *GhWRKY68* might be involved in developmental regulation. In addition, GUS expression was induced by various treatments. Slight GUS staining was observed in the absence of stress (Fig. 3C (a)). However, GUS expression was strongly induced in the SAM, root and leaf after NaCl, PEG, ABA or H_2_O_2_ treatments (Fig. 3C (b-e)). Taken together, these results suggest that *GhWRKY68* is a stress-inducible gene, and its expression is regulated spatially and temporally.

### The transcriptional levels of *GhWRKY68* are infiuenced by various stresses

**Fig 4:**
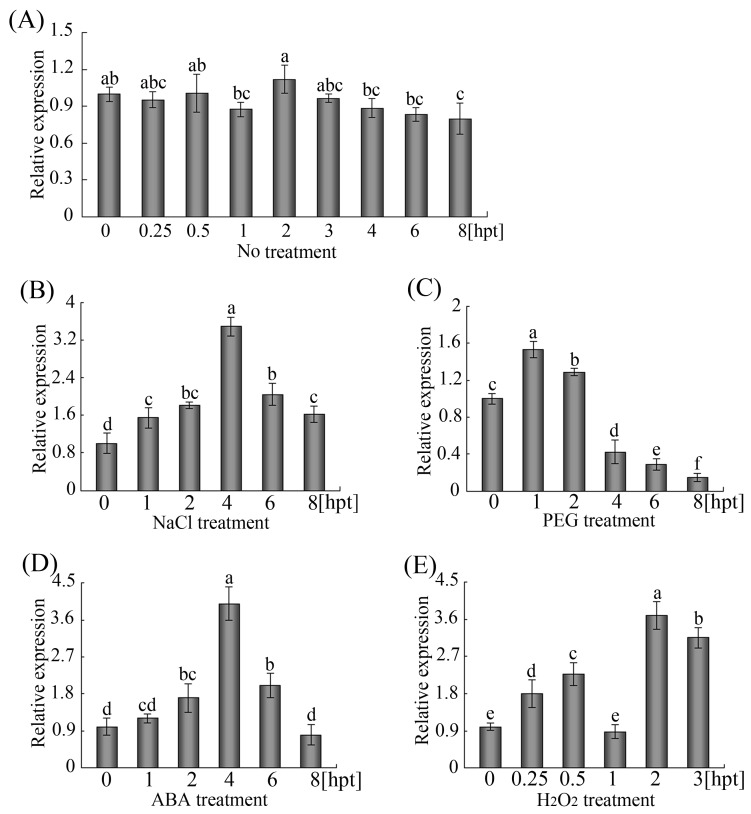
Expression profiles of *GhWRKY68* in cotton leaves under various stresses. Total RNA from cotyledons of 7-day-old cotton seedlings exposed to (A) no treatment (B) 200 mM NaCl, (C) 15% PEG6000, (D) 100 μM ABA, and (E) 10 mM H_2_O_2_ were obtained for qPCR. The lanes represent various times during the treatments. The expression profile of cotyledons that did not receive treatment was used as control. *GhUBQ* was included as an internal control, and the experiment was repeated at least twice.

Transcriptional modulation is a vital aspect of the complex signal transduction pathway that enables plants to respond to biotic and abiotic stresses [1, 3]. To study the expression patterns of *GhWRKY68* under diverse environmental stresses, transcript levels of this gene were measured after the cotton seedlings had been exposed to drought (PEG6000) and salt (NaCl). The expression profile of *GhWRKY68* in plants that were not exposed to treatment was used as control, and no changes in *GhWRKY68* transcript levels were noted during the 0- to 8-h series (Fig. 4A). As shown in Fig. 4B, treatment with NaCl strongly induced the transcription of *GhWRKY68* a 3.8-fold induction was observed at 4 hours post-treatment (hpt). The PEG6000 treatment also induced the expression of *GhWRKY68*, but the induced levels were lower than after NaCl treatment (Fig. 4C).

ABA and H_2_O_2_ are important signalling molecules that play crucial roles in the mediation of the expression of downstream genes in plant defence reactions against biotic and abiotic stresses [37]. To characterise the function of *GhWRKY68* in plant defences, we examined the expression pattern of *GhWRKY68* in cotton treated with various phytohormones using qPCR. In response to ABA, the *GhWRKY68* transcript levels increased within 1 hpt to 4 hpt, reaching maximal levels at 4 hpt (4.1-fold relative to mock-treated, Fig. 4D). The *GhWRKY68* transcript level was enhanced by H_2_O_2_: a 3.7-fold induction was observed at 2 hpt (Fig. 3E). These results indicate that *GhWRKY68* expression was induced under various stress conditions.

### Overexpression of *GhWRKY68* enhances the drought sensitivity of transgenic plants

The promoter analysis and differential expression patterns analysis suggested that *GhWRKY68* may play a role in multiple stress defence responses, especially in the osmotic stress response. Further functional analyses of *GhWRKY68* were performed through ectopic expression in *N*. *benthamiana* because the transformation of cotton plants is difficult and time consuming. Six independent transgenic *N*. *benthamiana* lines overexpressing (OE) *GhWRKY68* were obtained by kanamycin resistance selection and confirmed by PCR (S1 Fig. A), and the efficiency of tobacco transformation was 54.5%. RT-PCR and qPCR analyses were performed to detect the expression levels of the transgene in different lines (S1 Fig. B-C). Three representative T_2_ lines, OE1 (7#), OE2 (8#), and OE3 (10#), showing similar expression levels of *GhWRKY68*, were chosen for further experiments No phenotypic differences were observed between the *GhWRKY68*-OE transgenic lines and wild-type (WT) plants at any time during the life cycle.

**Fig 5:**
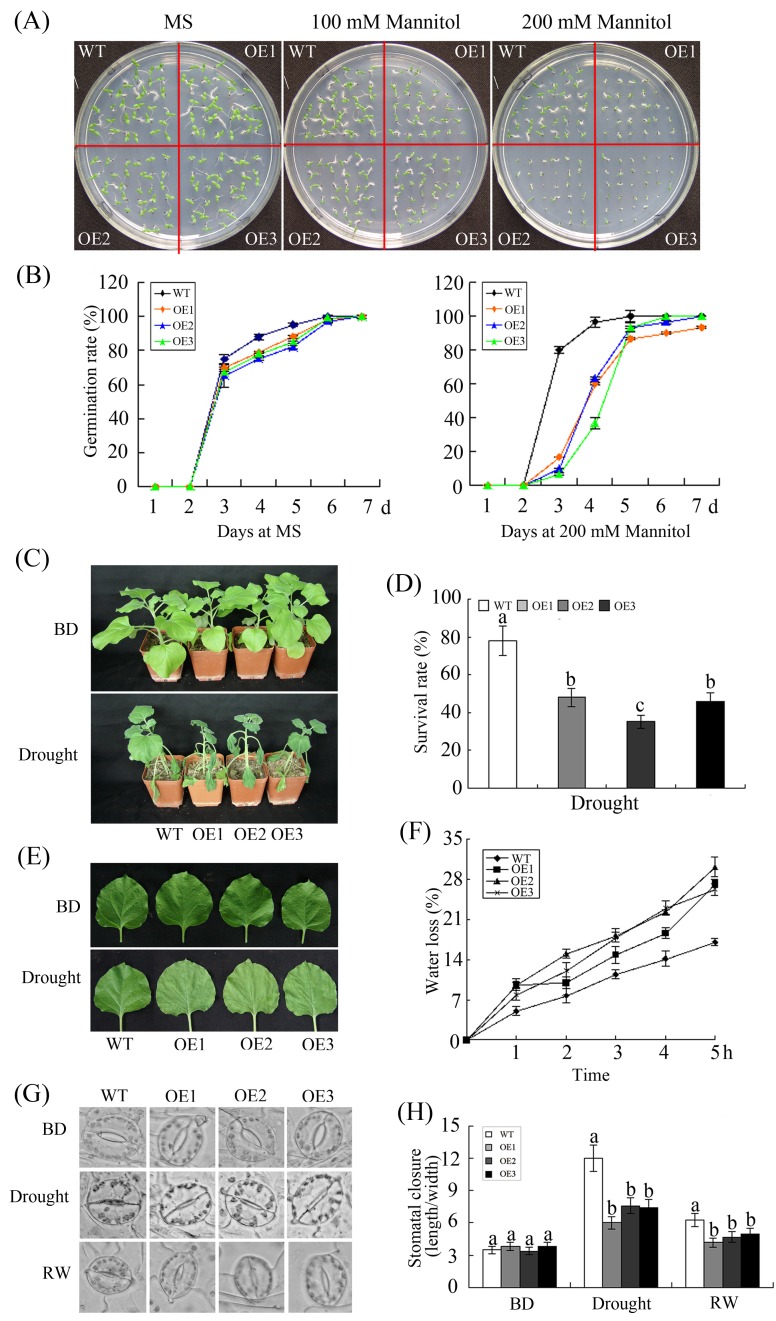
Enhanced drought sensitivity of transgenic *N*. *benthamiana* overexpressing *GhWRKY68*. (A) Seed germination on 1/2 MS medium with 0, 100, or 200 mM mannitol. (B) The germination rates of the WT and OE lines in 0 or 200 mM mannitol treatment conditions are presented. Germination was scored daily. (C) Representative phenotypes of 8-week-old WT and OE plants grown in soil before or after 10 days of drought. (D) Survival rates of WT and OE plants under drought conditions. (E) Representative phenotypes of the detached leaves of WT and OE plants under dehydration conditions. (F) Relative water loss rates from detached leaves of WT and OE plants were measured after dehydration at the indicated times. (G-H) Stomatal changes observed with a microscope before and after drought treatment. The stomatal length: width ratio is displayed. BD, before drought treatment; RW, re-watering. The data presented are the means ± SE of three independent experiments (n = 3). Different letters above the columns in (D) and (H) indicate significant differences (P < 0.0001) according to Duncan’s multiple range test performed using SAS version 9.1 software.

To determine the infiuence of drought on the transgenic lines, the germination capacity of WT and OE plants was evaluated on 1/2 Murashige & Skoog (MS) medium supplemented with exogenous mannitol (0, 100 and 200 mM) to mimic drought conditions. As shown in Fig. 5A-B, there were no significant differences in the growth or germination rates of the WT and OE plants during germination under normal conditions. However, following treatment with mannitol, the germination of both the WT and OE lines was inhibited as a function of increasing mannitol concentration. In addition, transgenic seeds were more strongly suppressed than wild-type seeds, resulting in the germination of the OE plants being approximately 10–15% of that of WT plants in the presence of 200 mM mannitol 3 days after sowing (Fig. 5A-B).

To further assess the effect of *GhWRKY68* overexpression on drought tolerance at the vegetative growth stage, 8-week-old WT and OE plants were grown in the same pot without water for 10 days. After 10 days of drought treatment, OE plants showed more leaf wilting than WT plants (Fig. 5C). When they were re-watered, the survival of the transgenic plants was approximately 30–43% lower than that of the WT plants (Fig. 5D). Additionally, the rate of water loss from the detached leaves of the OE plants was lower than that of WT plants under dehydration conditions (Fig. 5E-F). Stomatal closure is a major plant mechanism for reducing water loss during drought [38]. Thus, the stomatal state was observed by microscopy under drought conditions. Under normal conditions, there was no significant difference between the stomatal length:width ratios of the opened stomata of the WT and OE plants. However, the stomatal apertures in the OE lines were more open than those of the WT plants after drought stress. After a 2-day watering recovery, the stomata reopened, and the OE lines showed a higher length: width ratio than the WT plants (Fig. 5G-H). All our data indicate that the overexpression of *GhWRKY68* can enhance drought sensitivity in transgenic tobacco plants at both the seedling and the vegetative growth stages.

### The overexpression of *GhWRKY68* decreased tolerance to salt stress in transgenic plants

**Fig 6:**
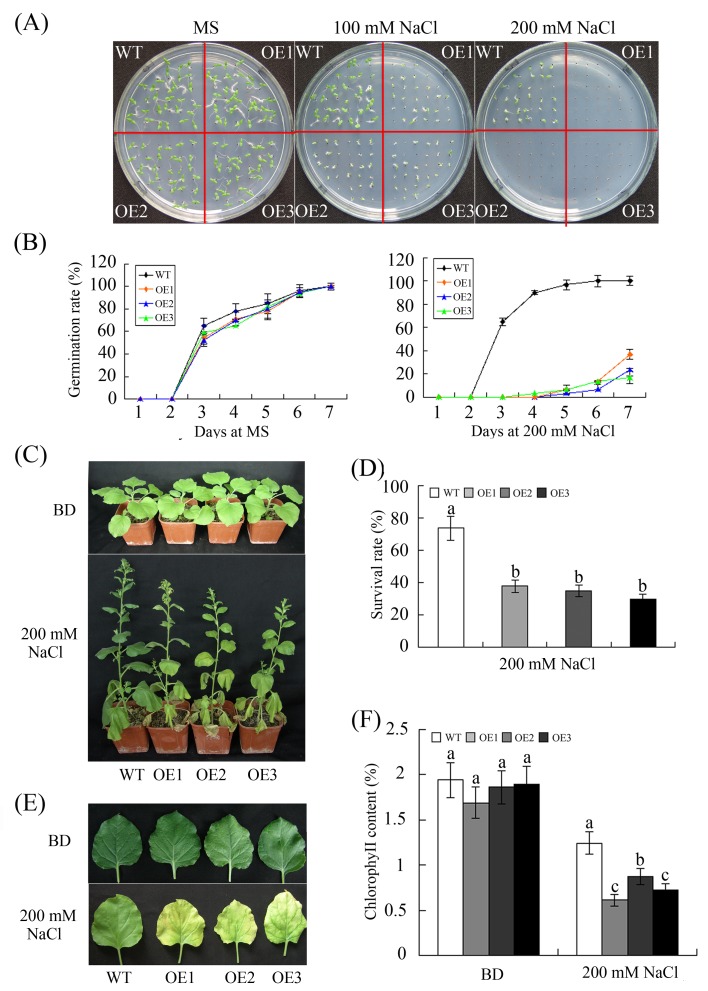
Reduced salt tolerance in transgenic plants overexpressing *GhWRKY68*. (A) Seed germination on 1/2 MS medium containing different concentrations of NaCl. (B) Germination rates of the WT and OE lines under 0 or 200 mM NaCl treatment conditions. Germination was scored daily. (C) Representative phenotypes of 8-week-old WT and OE plants grown in soil before and after 200 mM NaCl treatment for 1 month. (D) Survival rates of WT and OE plants after 200 mM NaCl treatment. (E) Representative phenotypes of the detached leaves of WT and OE plants treated with 200 mM NaCl. (F) Quantification of chlorophyll content. The values represent the chlorophyll content in the salt-treated plants relative to that of the untreated plants. BD, before drought treatment. The data presented are the means ± SE of three independent experiments (n = 3). Different letters above the columns in (D) and (F) indicate significant differences (P < 0.05) according to Duncan’s multiple range test performed using SAS version 9.1 software.

**Fig 7:**
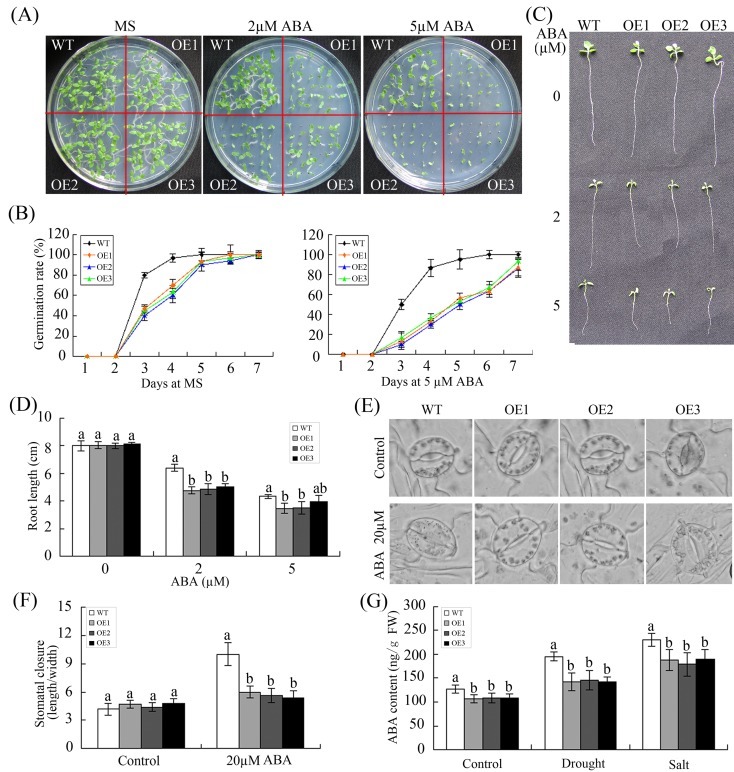
Overexpression of *GhWRKY68* negatively regulates ABA signalling in transgenic plants. (A) Seed germination of WT and OE lines on 1/2 MS medium containing different ABA concentrations (0, 2 or 5 μM). (B) Germination rates of the WT and OE lines under 0 or 5 μM ABA treatment conditions. Germination was scored daily. (C) Root elongation of WT and OE plants after treatment with different ABA concentrations (0, 2 or 5 μM). (D) Statistical analysis of the root length. (E) Micrographs showing the stomatal closure of transgenic and WT plants with or without 20 μM ABA treatment. (F) Stomatal closure response to ABA treatment. The data represent the means ± SE of 40 stomata from three independent experiments. (G) ABA contents of WT and OE plants before and after treatment. For drought treatment, 8-week-old OE and WT plants were grown in soil without water for 10 days. For salt treatment, WT and OE plants were irrigated with salt water (200 mM) for 1 month. The values represent the means ± SE of three independent experiments. The different letters above the columns indicate significant differences (*P* < 0.05) according to Duncan’s multiple range test.

To investigate the possible effects on salt tolerance of the constitutive expression of *GhWRKY68* in transgenic plants, both WT and OE plants were challenged with salt stress during the germination and vegetative stages. Seeds of WT and OE plants were germinated on 1/2 MS agar medium containing different NaCl concentrations (0, 100, or 200 mM). As illustrated in Fig. 6A-B, the presence of salt significantly affected the germination rates of OE seeds. To verify whether *GhWRKY68* decreased the salt tolerance of the transgenic plants in the vegetative stage, WT and OE plants were irrigated with salt water (200 mM) for 1 month. The OE plants showed severe growth retardation (Fig. 6C) and serious leaf curling and chlorosis (Fig. 6E) compared with WT plants after NaCl treatment. Approximately 40% of the OE plants survived the high-salinity conditions, a survival rate lower than that of WT plants (Fig. 7D). Furthermore, the total chlorophyll content of the OE plants was significantly less than that of the WT plants. Taken together, these results indicated that the overexpression of *GhWRKY68* might confer reduced tolerance to salt stress in transgenic plants during seed germination and in the vegetative stage.

### *GhWRKY68* overexpression negatively regulates ABA signalling in transgenic plants

ABA is an important phytohormone regulating plant development and various stress responses, including osmotic stress responses [39]. The expression of *GhWRKY68* increased significantly in response to ABA, indicating that *GhWRKY68* is involved in ABA signalling. Thus, the sensitivity of WT and OE plants exposed to ABA was explored. As shown in Fig. 7A-B, OE seeds showed lower germination rates than WT seeds in medium supplemented with various ABA concentrations. The responses to ABA during the post-germination growth stage were also assessed. The seeds of WT and OE plants were germinated on 1/2 MS medium for 2 days and were then transferred to medium supplemented with different ABA concentrations (0, 2 or 5 μM). In the absence of exogenously applied ABA, there was no significant difference in the root growth of WT and OE plants. However, in the OE plants, root growth was significantly inhibited, and the taproot length in the OE plants was less than that in the WT plants upon treatment with different concentrations of ABA (Fig. 7C-D). ABA-mediated stomatal closure plays a key role in osmotic regulation. Thus, the stomata were analysed to investigate whether the overexpression of *GhWRKY68* affected the sensitivity of guard cells to ABA treatment. Without ABA treatment, no difference in the stomatal length:width ratio was observed between WT and OE plants. However, the OE plants showed a lower ratio than the WT plants after 20 μM ABA treatment (Fig. 7E-F). Drought and salt stresses can trigger ABA-dependent signalling pathways [40]. The OE plants contained lower levels of ABA than the WT plants before and after drought and salt treatment (Fig. 7G).

**Fig 8:**
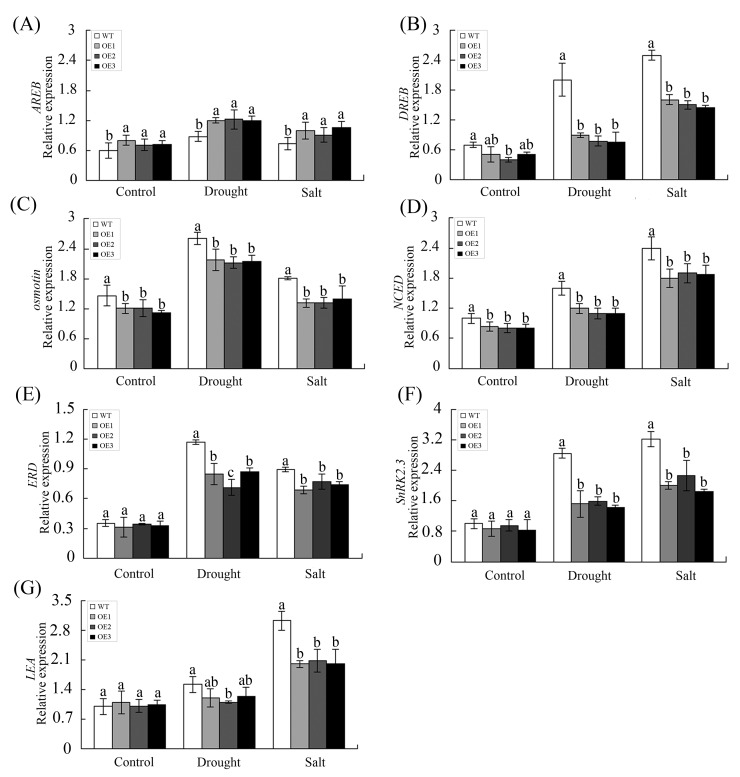
The expression of ABA-responsive genes under drought and salt stress conditions. (A) *AREB*, (B) *DREB*, (C) *osmotin*, (D) *NCED*, (E) *ERD*, (F) *SnRK*2.3, and *LEA* (G)correspond to the expression levels of these ABA-responsive genes in WT and OE lines analysed by qPCR. For drought treatment, 8-week-old OE and WT plants were grown in soil without water for 10 days. For salt treatment, WT and OE plants were irrigated with salt water (200 mM) for 1 month. The values represent the means ± SE of three independent experiments. The different letters above the columns indicate significant differences (*P* < 0.05) according to Duncan’s multiple range test.

To elucidate the possible mechanisms of *GhWRKY68-*mediated drought and salt sensitivity involving the ABA signalling pathway, we examined the expression of some ABA-responsive genes in the transgenic plants during drought and salt treatments. These genes included *NbAREB* (ABA-responsive element binding), *NbDREB* (dehydration-responsive element binding), *Nbosmotin*, and *NbNCED* (nine-cis-epoxycarotenoid dioxygenase), *NbERD* (early responsive to dehydration), *NbLEA* (late-embryogenesis-abundant protein), and *NbSnRK2*.*3* (SNF1-related protein kinase 2.3), which are stress-inducible marker genes that function in ABA-dependent and ABA-independent pathways [37, 41–44]. Under drought and salt stress, the expression levels of *NbDREB*, *Nbosmotin*, *NbNCED*, *NbERD*, *NbSnRK2*.*3* and *NbLEA* in the transgenic plants were reduced compared with their levels in WT plants (Fig. 8B-G); however, *NbAREB* transcript levels were increased in OE plants compared with WT plants (Fig. 8A).

### *GhWRKY68* mediates the accumulation of ROS and enhanced oxidative damage in transgenic plants

**Fig 9:**
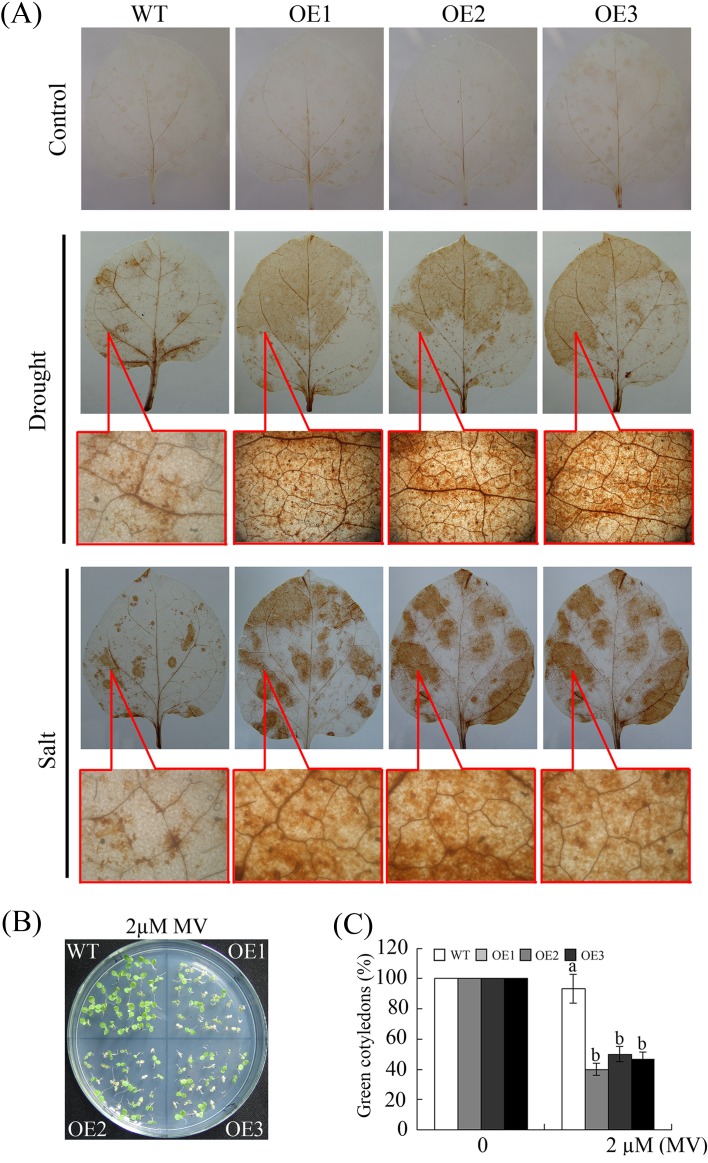
The constitutive expression of *GhWRKY68* increased ROS production and oxidative damage in transgenic plants. (A) Representative photographs of H_2_O_2_ accumulation using DAB staining. For drought and salinity treatments, the top figures show H_2_O_2_ accumulation, and the bottom figures illustrate the microscopic observations of the brown precipitate. (B) Phenotypes of young seedlings grown on medium containing 2 μM MV. (C) Quantification of cotyledon greening in the seedlings described in (B). The data represent the means ± SE of three independent experiments. The different letters above the columns in (C) indicate significant differences (*P* < 0.05) according to Duncan’s multiple range test performed using SAS version 9.1 software.

ROS act as important signal transduction molecules, mediating tolerance to various stresses, mainly O_2_^-^ and H_2_O_2_ [45]. Drought and salinity impose osmotic stress, which induces ROS production and causes ROS-associated injury [30, 46]. To investigate the tolerance to oxidative stress in the transgenic plants, the accumulation of O_2_^-^ and H_2_O2 in detached leaves from the WT and OE plants was measured using DAB staining. Untreated WT and OE plants were used as controls. As shown in Fig. 9A, no obvious O_2_^-^ or H_2_O_2_ accumulated in WT or OE plants under normal conditions. However, after treatment with drought and salt, the leaves of the OE lines accumulated higher levels of O_2_^-^ and H_2_O_2_ compared with WT leaves. Furthermore, microscopic analysis of the DAB staining revealed higher H_2_O_2_ accumulations in the leaves of OE plants than in the leaves of WT plants.

Methyl viologen (MV) is an herbicide that causes chlorophyll degradation and cell membrane leakage through ROS production [47]. This compound was used to examine the potential role of *GhWRKY68* in oxidative stress. As shown in Fig. 9B and 9C, in plants grown on medium containing 2 μM MV, more severe damage and significantly lower cotyledon greening rates occurred in OE plants than in WT plants. These results suggest that the overexpression of *GhWRKY68* increases MV-induced oxidative damage during the germination phase.

### The molecular mechanism by which *GhWRKY68* overexpression decreases oxidative stress tolerance in transgenic plants

**Fig 10:**
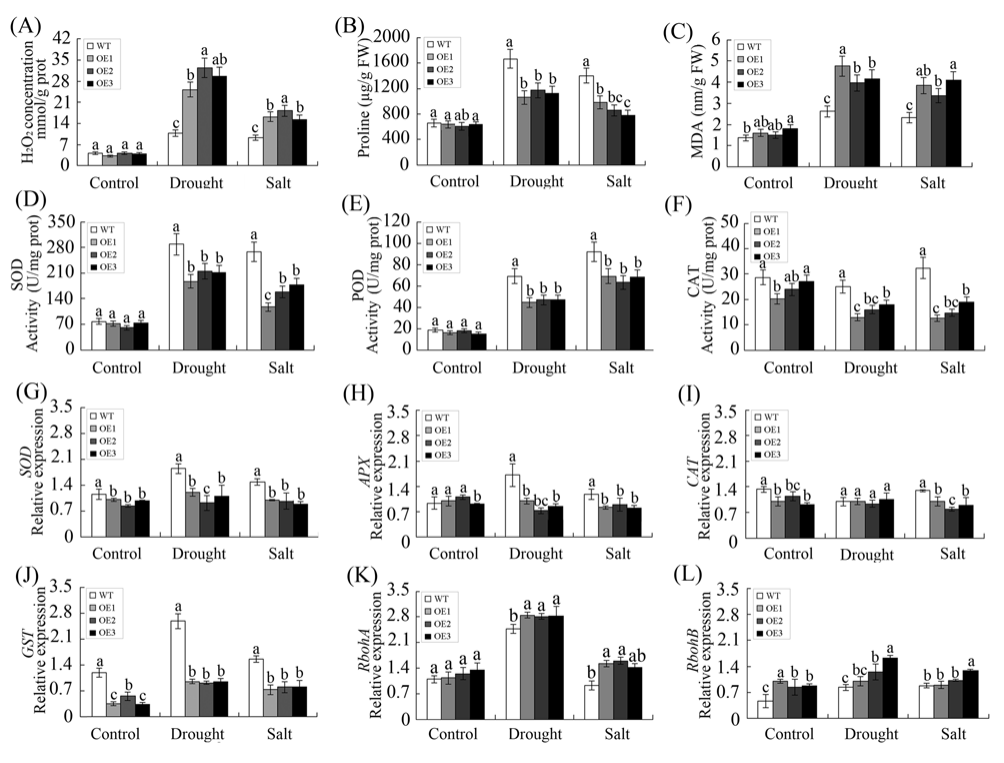
*GhWRKY68* overexpression decreased oxidative stress tolerance in transgenic plants under drought and salt stress conditions. (A) H_2_O_2_ concentration in WT and OE plants under drought and salt treatment. (D) Proline content in WT and OE plants under drought and salt treatment. (C) MDA content in WT and OE plants under drought and salt treatment. (D), (E) and (F) represent SOD, POD and CAT activities, respectively. (G) *SOD*, (H) *APX*, (I) *CAT*, (J) *GST*, (K) *RbohA* and (L) *RbohB* expression levels indicative of ROS-scavenging or ROS-producing genes in WT and OE lines analysed by qPCR. For drought treatment, 8-week-old OE and WT plants were grown in soil without water for 10 days. For salt treatment, WT and OE plants were irrigated with salt water (200 mM) for 1 month. The values represent the means ± SE of three independent experiments. The different letters above the columns indicate significant differences (*P*<0.05) based on Duncan’s multiple range tests.

To explore the possible mechanisms underlying the decreased tolerance of oxidative stress, several physiological indexes, including antioxidant enzyme activity and the expression of oxidation-related genes, were examined. The basal levels of H_2_O_2_, proline and malondialdehyde (MDA) in WT and OE plants were not different in normal conditions (Fig. 10A-C). Under drought and salt stress, the H_2_O_2_ and MDA contents were dramatically increased in the OE lines compared to the WT plants. In addition, drought and salt stress markedly increased the proline content in the leaves of the WT and OE plants, but the proline level was higher in WT plants than in the OE plants.

Antioxidative systems play crucial roles in regulating the intracellular ROS balance [48]. The potential role of *GhWRKY68* in oxidative stress was further evaluated by measuring antioxidant enzymatic activities. Three significant antioxidant enzymes, superoxide dismutase (SOD), peroxidase (POD) and catalase (CAT) were monitored before and after treatment (Fig. 10D-F). Under normal growth conditions, the activities of the three antioxidant enzymes were not different in the WT and OE plants. After the drought and salt treatments, the activities of SOD and POD were greatly increased in the WT and OE plants, and the WT plants showed significantly higher SOD and POD activity than the OE plants (Fig. 10D-E). The WT plants displayed a slight increase, and the OE plants displayed a significant decrease in CAT activity after the drought and salt treatment (Fig. 10F).

Furthermore, the transcript levels of several important ROS-related genes were measured by qPCR in WT and OE plants after drought and salt treatment (Fig. 10G-L). The genes chosen included *SOD*, *APX*, *CAT* and *GST*, which encode ROS-scavenging enzymes, and the respiratory burst oxidase homolog genes (*RbohA* and *RbohB*), which encode ROS producers. After drought and salt treatment, the expression patterns of *SOD*, *APX* and *CAT* were only slightly altered in the OE lines, and the transcript accumulation was lower than in WT plants (Fig. 10G-I). However, the expression of *GST* was increased in the OE plants, although it remained lower than in WT plants (Fig. 10J). In addition, the expression levels of *RbohA* and *RbohB* were markedly elevated in the OE plants and were significantly higher than in the WT plants (Fig. 10K-L).

## Discussion

Although many *WRKY* genes have been studied over the last decade, our understanding of the regulation and action of the gene family is relatively limited [3]. To date, few *WRKY* genes have been functionally characterised in cotton (*Gossypium hirsutum*). In the present study, we report, for the first time, a novel group IIc WRKY gene, *GhWRKY68*, which encodes a nuclear-localised protein that specifically binds to the W-box [TTGAC(C/T)] and transactivates the expression of downstream GUS reporter genes in plant leaves. Our results suggest that overexpression of *GhWRKY68* in *Nicotiana benthamiana* remarkably reduces the plants’ tolerance to drought and salt stresses through ABA signalling and the regulation of cellular levels of ROS.

Numerous studies have suggested that the regulation of TFs is highly complex, involving transcript and protein levels, DNA binding, subcellular localisation, and other properties through posttranslational mechanisms [3]. The subcelluar localisation analysis demonstrated that the GhWRKY68 protein localised to the nucleus (Fig. 2B). Furthermore, a yeast one-hybrid (Fig. 2D) and transient co-expression experiment (Fig. 2F) demonstrated that *GhWRKY68* specifically binds to the W-box [TTGAC(C/T)] and functions as a transcriptional activator. These observations suggest that *GhWRKY68* may activate the expression of target genes in the nucleus and may participate in various plant processes, forming a network with other genes by binding to the W-box [TTGAC(C/T)] in the promoters of defence-associated genes as well as many WRKY genes [2, 3].

Growing evidence has shown that WRKY proteins are involved in plant responses to various abiotic stresses [13–15]. For example, *WRKY70* and *WRKY54* are negative regulators that modulate osmotic stress tolerance in *Arabidopsis* [12]. Transcript levels of *DgWRKY1* were increased by drought and salt stress in *Chrysanthemum* [49]. In addition, Wang *et al* [50] reported that 15 *VvWRKY*s are involved in the low temperature-related signalling pathways in grapes. We found that *GhWRKY68* transcripts can be induced by abiotic stresses (PEG and NaCl) and by multiple defence-related signalling molecules (ABA and H_2_O_2_) (Fig. 4). Consistent with these observations, GUS staining analyses (Fig. 3C) of drought and salt stressed *GhWRKY68*-overexpressing plants (Fig. 5–6) revealed that *GhWRKY68* plays a role in drought, salt, ABA and ROS stress tolerance, and we speculated that the role of *GhWRKY68* in the plants’ response to drought and salt stress depends on modulating ABA signalling and regulating cellular ROS.

ABA is an important phytohormone that inhibits seed germination and seedling growth [51] and mediates plant development and response to various stresses [39]. Drought and salt stresses can induce ABA accumulation by triggering ABA-dependent signalling pathways [40], and an increased ABA content is beneficial for plants under stress conditions [39]. In our study, seed germination and root growth of *GhWRKY68*-overexpressing plants were significantly inhibited by exogenous ABA compared with WT plants (Fig. 7A-D). The *GhWRKY68* transgenic plants were less sensitive to ABA-induced stomata closure (Fig. 7E-F). Moreover, the OE lines accumulated less ABA during drought and salt stress compared with the WT plants (Fig. 7G). All these results indicated that *GhWRKY68* might confer reduced drought and salt tolerance by negatively regulating the ABA pathways. Consistent with these results, *DgWRKY1* was involved in the ABA-dependent signalling pathway under salt stress conditions [49].

Under stress conditions, TFs play important roles by regulating the expression of target genes to enhance plant stress tolerance [3]. For example, *ThWRKY4* activates many genes, including *ARR15*, *ATCTH*, *EPR1* and *ARR6*, which were previously reported to be involved in stress tolerance [52]. In this study, *GhWRKY68* responded to drought and salt stresses by modulating ABA signalling. Like some other *WRKYs*, *GhWRKY68* can also regulate the expression of the ABA-responsive genes *AREB*, *DREB*, *osmotin*, *ERD*, *SnRK2*.*3*, *LEA* and *NCED*, which have been reported to function in the ABA-dependent or ABA-independent pathways [11, 41–44]. In the ABA-dependent pathway, AREB serves as a major ABA-responsive element that can bind and activate the expression of AREB-binding genes. *DREB* is known to regulate the expression of many stress-inducible genes in the ABA-independent pathways. *Osmotin* is responsive to ABA and is involved in the adaptation to low water potential [53]. *ERD*, *SnRK2*, and *LEA* genes were target genes of *AREB* or *DREB* genes [37, 41, 43, 44], which may contain W-box elements in their promoters and be recognized by interacting WRKY proteins through formation of a DNA loop to regulate many genetic processes including transcription regulation [3]. The *NCED* gene encodes 9-cis-epoxycarotenoid dioxygenase, a key enzyme of ABA biosynthesis and a known a participant in ABA mediated responses [54]. As shown in Fig. 8A-G, under drought and salt stresses, *NbAREB* was up-regulated, but *NbDREB*, *Nbosmotin*, *NbNCED*, *NbERD*, *NbSnRK*2.3, and *NbLEA* were down-regulated in transgenic plants compared with WT plants. In summary, it is likely that *GhWRKY68* is involved in drought and salt stress through ABA-dependent and ABA-independent signalling pathways.

ROS mainly consist of O_2_^-^ and H_2_O_2_ and can be induced in plants by drought and salt stresses [30, 46]. The ROS level is critical for abiotic stress tolerance in plants [31]. Overproduction of H_2_O_2_ can kill the leaf cells and cause leaf necrosis in plants [55]. Proline contributes to osmotic adjustment and protects macromolecules during dehydration, acting as both an osmotic agent and a radical scavenger. The accumulation of proline may participate in scavenging ROS in response to stress [56, 57]. MDA is the final decomposition product of lipid peroxidation, and the level of MDA reflects the degree of plant damage [58]. In the present study, under drought and salt stresses, the overexpression of *GhWRKY68* enhanced ROS accumulation, reduced the proline content (Fig. 10B) and elevated the MDA content (Fig. 10C). In plants, the most common mechanism for oxidative tolerance is the regulation of the ROS-scavenging enzymes [59]. A subsequent study revealed that the activities of SOD and POD in the *GhWRKY68*-overexpressing plants were lower than those in the WT plants during drought and salt stress (Fig. 10D-E). Furthermore, after drought and salt treatments, the transcript levels of the ROS-related genes *SOD*, *APX*, *CAT* and *GST* were lower, and the activities of SOD, POD and CAT decreased (Fig. 10G-L). In addition, the expression levels of *RbohA* and *RbohB* were significantly higher than in WT plants (Fig. 10K-L), resulting in the increase of O^2-^ and H_2_O_2_. The increased levels of O^2-^ and H_2_O_2_ were due, at least in part, to the decreased stress tolerance in transgenic plants. Briefly, these results suggest that *GhWRKY68* might contribute to drought and salt stress by regulating ROS pathways and that the role of *GhWRKY68* in the ROS scavenging pathway is complex.

Over the past several years, a substantial number of WRKYs have been shown to participate in protein—protein interactions, and complex functional interactions have been observed between WRKY proteins and other regulatory proteins (such as MAPKs, VQ proteins, chromatin remodeling proteins, histone deacetylases, 14-3-3 proteins and calmodulin) involved in the modulation of important biological processes [3, 4]. For example, *Arabidopsis* MEKK1 directly interacts with the senescence-related WRKY53 transcription factor at the protein level [60]. In addition, approximately 50% of the VQ proteins interact with the group IIc AtWRKY51 [61]. AtWRKY7 and 10 additional *Arabidopsis* Group IId WRKY proteins can bind to calmodulin (CaM), which is a Ca^2+^-binding signalling protein [62]. Moreover, HDAC and histone proteins were recently identified as WRKY-interacting proteins [3]. Mapping of dynamic and complex protein—protein interactions in WRKY-mediated transcription of important target genes is critical to develop a comprehensive understanding of the WRKY signalling and transcriptional regulatory network [3].

In conclusion, *GhWRKY68* functions as a transcription factor that responds to drought and salt stress by modulating ABA signalling and the regulation of cellular ROS. The modulation of ABA-responsive genes and the activation of ROS-related antioxidant genes and enzymes were partially correlated. It has been reported that cellular ROS levels are regulated through the ABA-triggered regulation of ROS-producing and ROS-scavenging genes [46], but the mechanisms that control ROS signalling through ABA during drought and salt stress remain unclear. Meanwhile, combined with stress signals, *GhWRKY68* may regulate the downstream W-box-containing genes by binding to W-box motifs in the promoters of genes involved in ABA signalling, forming a network with other defence-associated genes. Thus, our findings not only extend knowledge regarding the biological function of the group IIc WRKY proteins but also provide new insights for further manipulation of crop plants to improve stress tolerance.

## Materials and Methods

### Plants materials and treatments

Cotton (*Gossypium hirsutum* L. cv. lumian 22) seeds were germinated and grown in greenhouse conditions at 25°C with a 16 h light/8 h dark cycle (light intensity of 200 μmol m^-2^ s^-1^; relative humidity of 60–75%). Seven-day-old cotton seedlings were subjected to different treatments. For the signalling molecule treatments, seedling leaves were sprayed with 100 μM ABA, or 10 mM H_2_O_2_ as described previously [63]. For the salt and drought treatments, the seedlings were cultured in solutions containing 200 mM NaCl or 15% (w/v) PEG6000. Seedlings without any treatment were used as controls. All the samples were frozen in liquid nitrogen at the appropriate time and stored at -80°C for RNA extraction. Each treatment was repeated at least twice.

*Arabidopsis thaliana* Columbia ecotype (Col-0) and transgenic *Arabidopsis* seeds were sown on 1/2 MS agar medium in a growth chamber at 22 ± 1°C with a 16/8 h light/dark cycle and a relative humidity of 80%. For the GUS assays, two-week-old transgenic *Arabidopsis* T_3_ seedlings were exposed to 100 μM ABA, 10 mM H_2_O_2_, 15% (w/v) PEG6000 or 200 mM NaCl. Additionally, *Nicotiana benthamiana* seeds were surface-sterilised and germinated on 1/2 MS agar medium under greenhouse conditions. Then, two-or three-leaf stage seedlings were transplanted into soil and maintained in greenhouse conditions.

### RNA extraction, cDNA synthesis and DNA preparation

An improved CTAB-ammonium acetate method was used for total RNA isolation from cotton according to the method described by Zhao et al [64]. Total RNA was digested with RNase-free DNaseI (Promega, USA) according to the manufacturer’s recommendations to remove the genomic DNA. Then, the RNA was used for first-strand cDNA synthesis with reverse transcriptase (TransGen Biotech, China) following the manufacturer's protocol. Genomic DNA was isolated from seedling leaves using the CTAB method described by Porebski et al [65].

### Gene isolation, vector construction and genetic transformation

The *GhWRKY68* cDNA and genomic sequences were isolated as described previously [34]. All of the primers used in this study are listed in S1 Table. Analysis of the amino acid sequence and the promoter sequence of *GhWRKY68* were performed Using DNAman version 5.2.2 (Lynnon Biosoft, Quebec, Canada) and PlantCARE. The coding region of the gene was inserted into the plant expression vector PBI121 under control of the CaMV 35S promoter. The genetic transformations with the recombinant plasmids and the production of the transgenic *N*. *benthamiana* plants were accomplished using the procedures of Zhang et al [66]. The *GhWRKY68* promoter fragment was fused to the GUS reporter gene in the pBI121 binary vector to construct the recombinant plasmid ProGhWRKY68::GUS. The transgenic *Arabidopsis* plants were obtained as described by Shi et al [67]. The transgenic T_3_ lines were used for GUS histochemical staining assays to analyse the promoter activity as described by Baumann et al [68].

### Quantitative real-time PCR

Quantitative real-time PCR (qPCR) was performed using the SYBR Premix Ex Taq (TaKaRa, Dalian, China) and the CFX96TM Real-Time PCR Detection System (Bio-Rad, Hercules, CA, USA). The PCR mix was composed of 10 μl SYBR Premix Ex Taq, 1.6 μl of 1:10 diluted cDNA, 0.4 μl of each primer (10 mM), and 7.6 μl PCR grade water in a final volume of 20 μl. The reactions were incubated under the following conditions: 1 cycle of 95°C for 30 sec; 40 cycles at 95°C for 5 sec, 55°C for 15 sec, and 72°C for 15 sec; and then a single melt cycle from 65 to 95°C. Each sample was analysed in triplicate, and the expression levels were calculated using the 2^-ΔΔCt^ comparative CT method [69]. Three independent experiments were performed. The primers used in qPCR are listed in S1 Table.

### Subcellular localisation analysis of GhWRKY68

To construct the 35S-GhWRKY68::GFP expression plasmid, the *GhWRKY68* coding region without the termination codon was inserted into the binary vector pBI121-GFP, which has a green fluorescence protein (GFP) gene driven by the Cauliflower mosaic virus (CaMV) 35S promoter. For transient expression, the recombined plasmid and the positive control 35S-GFP plasmid were transferred into living onion epidermal cells via the biolistic bombardment transformation method as described by Shi et al [67], using the Biolistic PDS-1000/He system (Bio-Rad, USA) with gold particles (1.0 μl) and a helium pressure of 1,350 psi. The fluorescence was observed using a confocal laser scanning microscope (LSM 510 META, ZEISS, Germany) after the tissues were stained with 100 μg/ml of 4’,6-diamidino-2-phenylindole (DAPI) (Solarbio, Beijing, China) in phosphate-buffered saline buffer for 10 min as described previously [34].

### Binding assays using the yeast one-hybrid system

A yeast one-hybrid assay was performed using the Matchmaker Gold Yeast One-Hybrid Library Screening System (Clontech, Palo Alto, CA) for the binding assay. According to the manufacturer’s protocol, the reporter vector pAbAi containing triple tandem copies of the W-box (TTGACC) was introduced into the yeast strain Y1HGold, forming a W-box-specific reporter strain used as bait. The pGAD-GhWRKY68 yeast expression vector was formed with the ORF of the *GhWRKY68* fused to the one-hybrid vector pGADT7 with the GAL4 activation domain. Then, pGADT7 and pGAD-GhWRKY68 were transformed into the W-box-specific reporter strain. The cells were plated on SD/-Leu/-Ura medium containing 500 ng/ml AbA to observe yeast growth. The 500 ng/ml AbA completely suppressed the basal expression of the pAbAi-W-box reporter strain in the absence of prey. Mutant W-box (mW-box) (TAGACG) was used as a negative control.

### Co-transfection experiments

The effector plasmid (35S:GhWRKY68) was constructed by inserting the *GhWRKY68* ORF into the binary vector pBI121 and replacing the GUS downstream from the CaMV35S promoter. For the reporter vector (W-box-35S mini-GUS plasmid), three tandem W-box sequences were fused to the CaMV 35S minimal promoter (W-box-35S mini), which substituted for the CaMV 35S promoter in pBI121GUS (Clontech). The effector and reporter plasmids were introduced into *Agrobacterium tumefaciens* strain GV3101. The *Agrobacterium*-mediated transient transformation assay was performed according to the method described by Yang et al [70].

### Analysis of transgenic plants under salt and drought conditions

For drought treatment, three T_3_ generation independent *GhWRKY68*-OE lines (OE1, OE2 and OE3) and wild-type seeds were surface sterilised and plated on 1/2 MS medium with different concentrations of mannitol (0, 100, or 200 mM), and the germination percentage was measured daily. Additionally, water was completely withheld for 10 days from 8-week-old OE and WT plants sown in soil, and the survival rates (the number of surviving plants relative to the total number of treated plants) were recorded after re-watering for 1 week. For the transpiration water loss assay, fully expanded leaves of OE and WT plants were detached and weighed immediately (fresh weight) with an electronic balance at room temperature, and the changes in fresh weight were recorded at designated times thereafter. The rate of water loss was calculated relative to the initial fresh weight. After the drought treatment, stomatal changes were observed by microscopy, and the ratio of stomatal length to width was recorded.

To examine salt tolerance, the seed germination percentage on 1/2 MS medium with different concentrations of NaCl (0, 100, or 200 mM) was measured by the method above. In addition, 8-week-old OE and WT plants were irrigated daily with 200 mM NaCl solution every day for 1 month and maintained under the same growth conditions as described above to record survival rates. Subsequently, the chlorophyll content was measured as described by Lichtenthaler and Wellburn [71]. The drought and salt stress analyses were repeated at least three times.

### ABA sensitivity analysis

To examine the response to ABA, the seeds were sown on 1/2 MS with different concentrations of ABA (0, 2, or 5 μM). The seed germination percentages and the root lengths were measured. In addition, a stomatal aperture assay was performed essentially as previously described [72, 73]. The stomatal apertures from the leaves of OE and WT plants treated with 5 μM ABA for 3 h were observed using a fiuorescence microscope (BX51 Olympus). The ratio of stomatal length to width indicated the degree of stomatal closure. For each treatment, at least 50 stomatal apertures were measured. Endogenous ABA was extracted as described previously [74], and the ABA content was measured using an ELISA kit (Fangcheng, Beijing, China) according to the manufacturer’s instructions.

### Oxidative stress analyses

For oxidative damage analyses, the seeds were germinated on 1/2 MS medium supplemented with 5 μM methyl viologen (MV), and the cotyledon greening rates were calculated. To detect the accumulation of H_2_O_2_ and O^2-^, a histochemical staining procedure was performed using 3, 3'- diaminobenzidine (DAB) according to the method described by Zhang et al [66, 70]. In addition, the H_2_O_2_ concentration and MDA content were determined using a hydrogen peroxide test kit and a maleic dialdehyde assay kit (Nanjing Jiancheng Bioengineering Institute), respectively, according to the manufacturer's instructions. The free proline content was monitored as described by Shan et al [75]. These experiments were repeated at least three times. Enzymes were extracted in a phosphate buffer (pH 7.8) and were quantified with the BCA Protein Assay Kit (Nanjing Jiancheng Bioengineering Institute). The antioxidant enzyme activities of superoxide dismutase (SOD), peroxidase (POD), and catalase (CAT) were measured with kits produced by the Nanjing Jiancheng Institute.

### Statistical analysis

The results are expressed as the mean ± standard deviation (SD) of triplicate experiments (n = 3). Statistical significance was determined by Duncan’s multiple range test with an analysis of variance (ANOVA) using Statistical Analysis System (SAS) version 9.1 (Version 8e, SAS Institute, Cary, NC, USA). The significance was set at P<0.05.

## Supporting Information

S1 Table Details of the primers used in this study. (DOC)

S1 Fig Identification of transgenic plants. (A) Evaluation of transgenic plants in the T_0_ progeny of transgenic plants by RT-PCR. (B) Analysis of *GhWRKY68* expression in wild-type (WT) and T_1_ OE plants.
(TIF)
