# Correction: *GhWRKY68* Reduces Resistance to Salt and Drought in Transgenic *Nicotiana benthamiana*

**DOI:** 10.1371/journal.pone.0213540

**Published:** 2019-03-12

**Authors:** Haihong Jia, Chen Wang, Fang Wang, Shuchang Liu, Guilin Li, Xingqi Guo

After publication of this article [1], concerns were raised about Figs 3, 5, 6, 7 and 9, and S1 Fig:

- Fig 3A was the ProGhWRKY68::GUS activity during different developmental stages including 2-week-old transgenic seedlings (Fig 3A (c)). Fig 3C was histochemical assays of GUS activity in response to various stresses using 2-week-old transgenic seedlings. To show the stresses treatments (Fig 3C) were dealing with 2-week-old transgenic seedlings, the authors used the same control figure as Fig 3A (c). The underlying data now provided are from the original experiment.
- For Fig 5A and Fig 6A, the authors used the same control because the germination rate assay under NaCl and mannitol treatments were conducted at the same time. The underlying data now provided are from the original experiment.
- For Fig 7E, the authors used the same image to represent WT and OE2 in the control line in error. The underlying data now provided are from the original experiment.
- For Fig 9A, the authors used the same microscopic observations of the brown precipitate to represent OE1 and OE2 in drought treatment and the same image to represent OE2 and OE3 in salinity treatment in error. The underlying data now provided are from the original experiment.
- For S1 Fig, the authors used the original image to make Panel B. The authors identified the transgenic plant using three methods. S1A and S1C Fig supported the result of S1B Fig, and the original data underlying panels A, B, C have been provided. The revised S1 Fig is from a replication experiment conducted after the publication of this article.

In addition, the primary data underlying results in this article were not included with the published article, although the Data Availability Statement for this article stated, “All relevant data are within the paper and its Supporting Information files.” With this Correction, the authors provide the original raw data via the

Harvard Dataverse at https://doi.org/10.7910/DVN/UOR2A7.

A member of *PLOS ONE*’s Editorial Board confirms that the revised figures and the raw data support the results and conclusions of the published article. The authors apologize for the errors in the published article.

The authors have provided corrected Figs 3, 6, 7 and 9 here.

**Fig 3:**
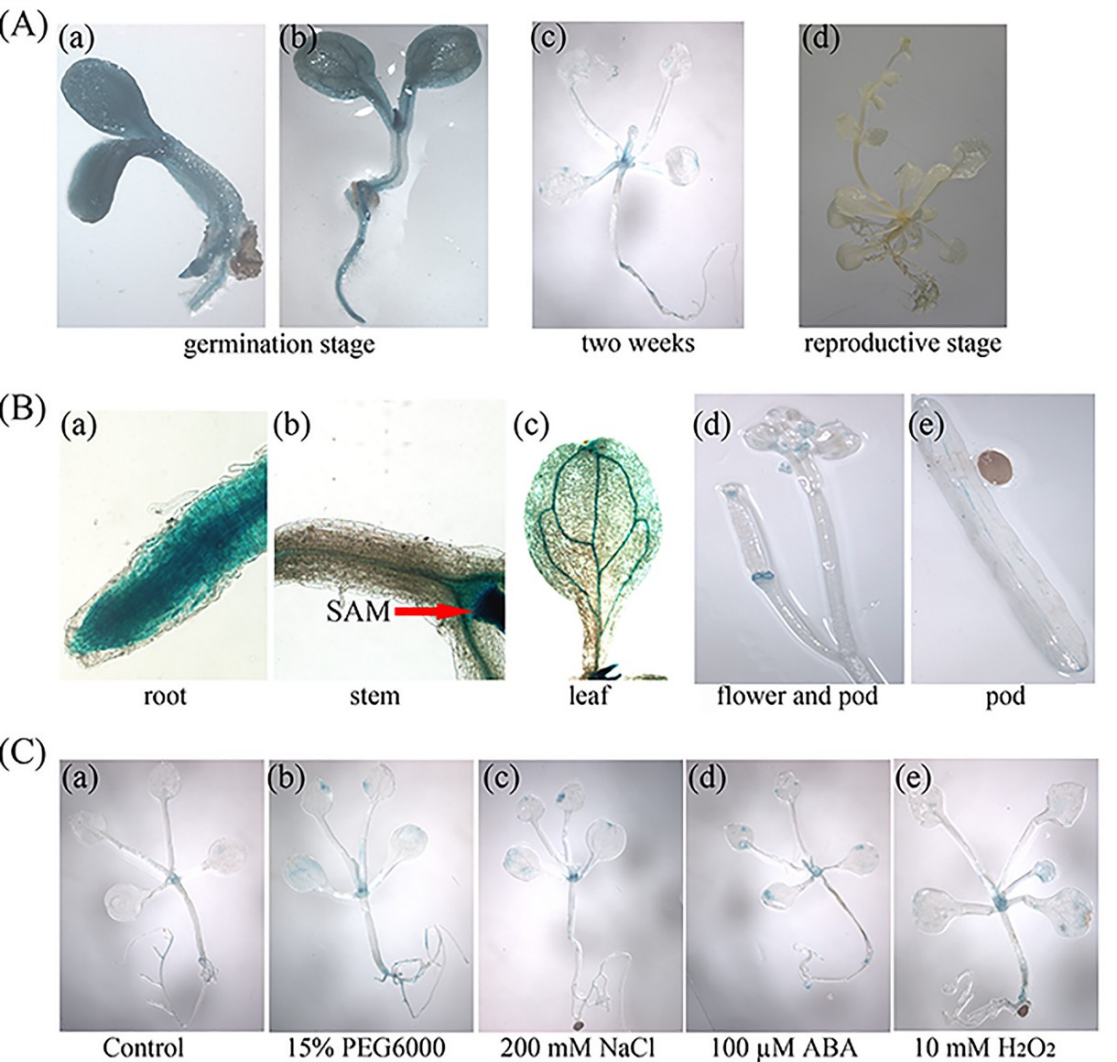
Histochemical analyses of GUS activity in ProGhWRKY68::GUS transgenic *Arabidopsis*. (A) ProGhWRKY68::GUS activity during different developmental stages. (B) Activity of the ProGhWRKY68::GUS construct in different tissue regions. (C) Histochemical assays of GUS activity in response to various stresses.

**Fig 6:**
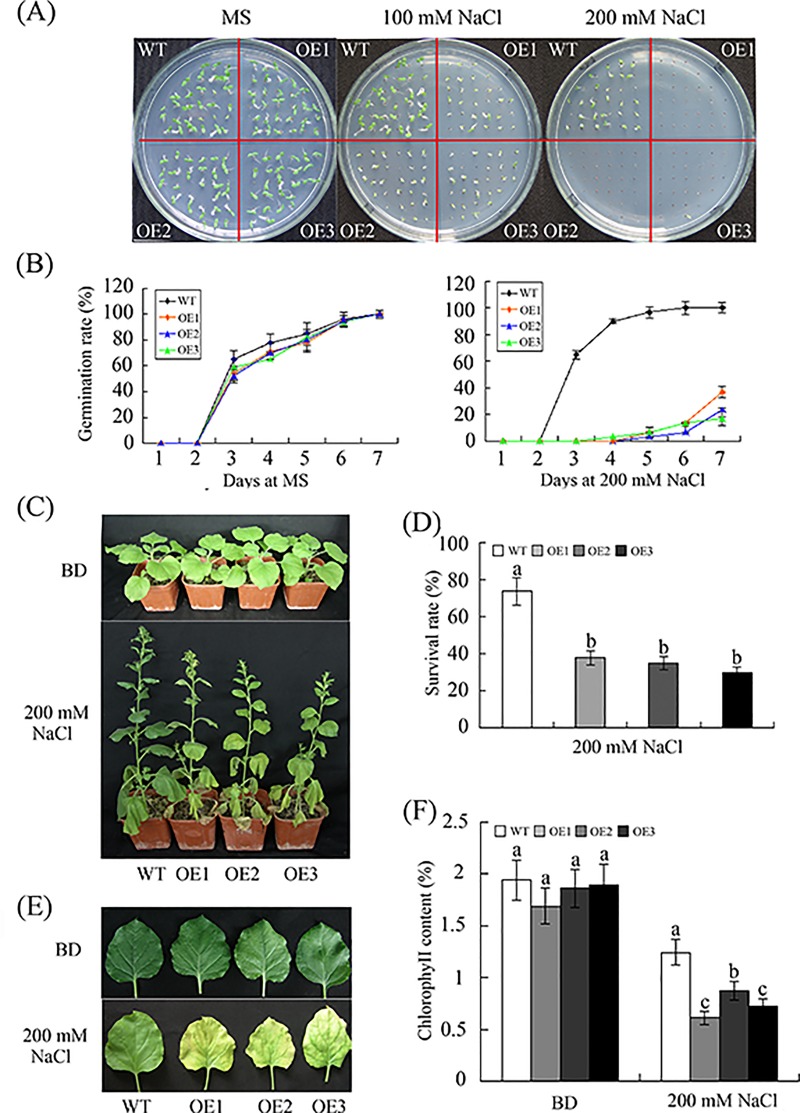
Reduced salt tolerance in transgenic plants overexpressing *GhWRKY68*. (A) Seed germination on 1/2 MS medium containing different concentrations of NaCl. (B) Germination rates of the WT and OE lines under 0 or 200 mM NaCl treatment conditions. Germination was scored daily. (C) Representative phenotypes of 8-week-old WT and OE plants grown in soil before and after 200 mM NaCl treatment for 1 month. (D) Survival rates of WT and OE plants after 200 mM NaCl treatment. (E) Representative phenotypes of the detached leaves of WT and OE plants treated with 200 mM NaCl. (F) Quantification of chlorophyll content. The values represent the chlorophyll content in the salt-treated plants relative to that of the untreated plants. BD, before drought treatment. The data presented are the means ± SE of three independent experiments (n = 3). Different letters above the columns in (D) and (F) indicate significant differences (P < 0.05) according to Duncan’s multiple range test performed using SAS version 9.1 software.

**Fig 7:**
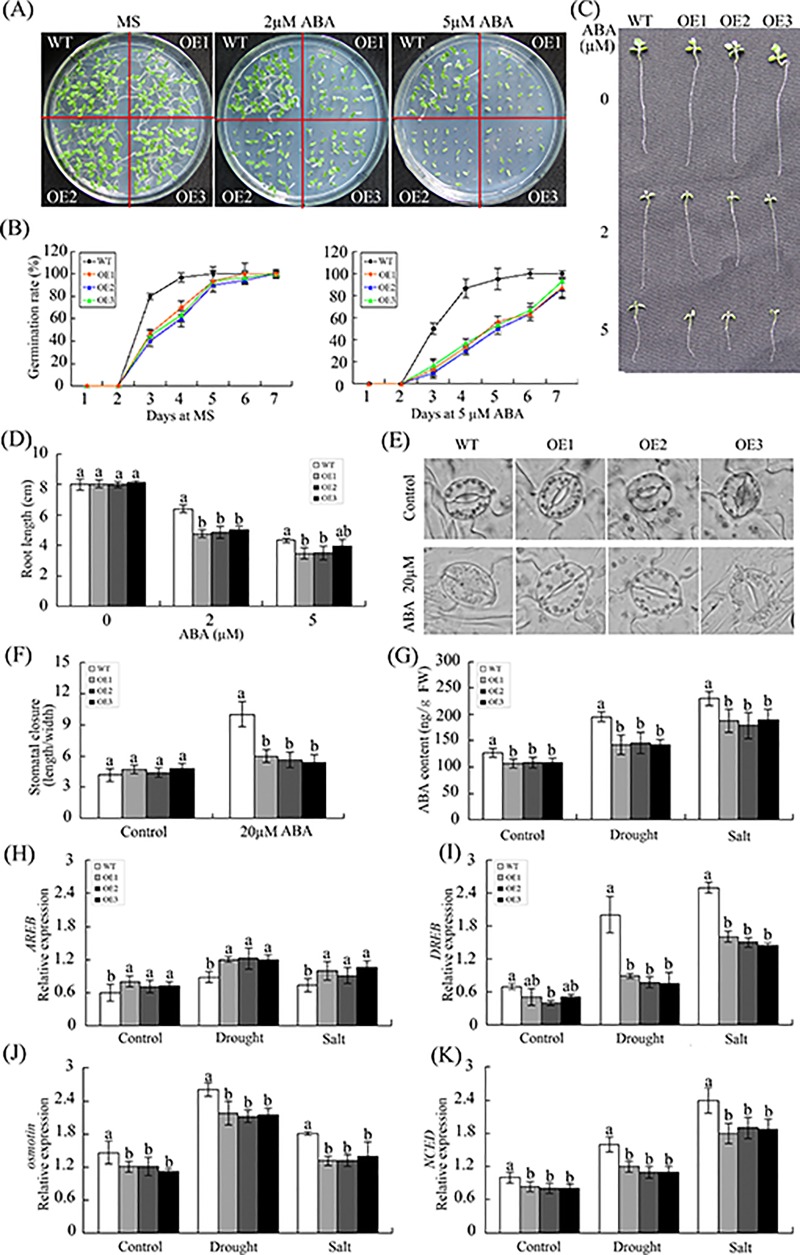
Overexpression of *GhWRKY68* negatively regulates ABA signalling in transgenic plants. (A) Seed germination of WT and OE lines on 1/2 MS medium containing different ABA concentrations (0, 2 or 5 μM). (B) Germination rates of the WT and OE lines under 0 or 5 μM ABA treatment conditions. Germination was scored daily. (C) Root elongation of WT and OE plants after treatment with different ABA concentrations (0, 2 or 5 μM). (D) Statistical analysis of the root length. (E) Micrographs showing the stomatal closure of transgenic and WT plants with or without 20 μM ABA treatment. (F) Stomatal closure response to ABA treatment. The data represent the means ± SE of 40 stomata from three independent experiments. (G) ABA contents of WT and OE plants before and after treatment. For drought treatment, 8-week-old OE and WT plants were grown in soil without water for 10 days. For salt treatment, WT and OE plants were irrigated with salt water (200 mM) for 1 month. The values represent the means ± SE of three independent experiments. The different letters above the columns indicate significant differences (*P* < 0.05) according to Duncan’s multiple range test.

**Fig 9:**
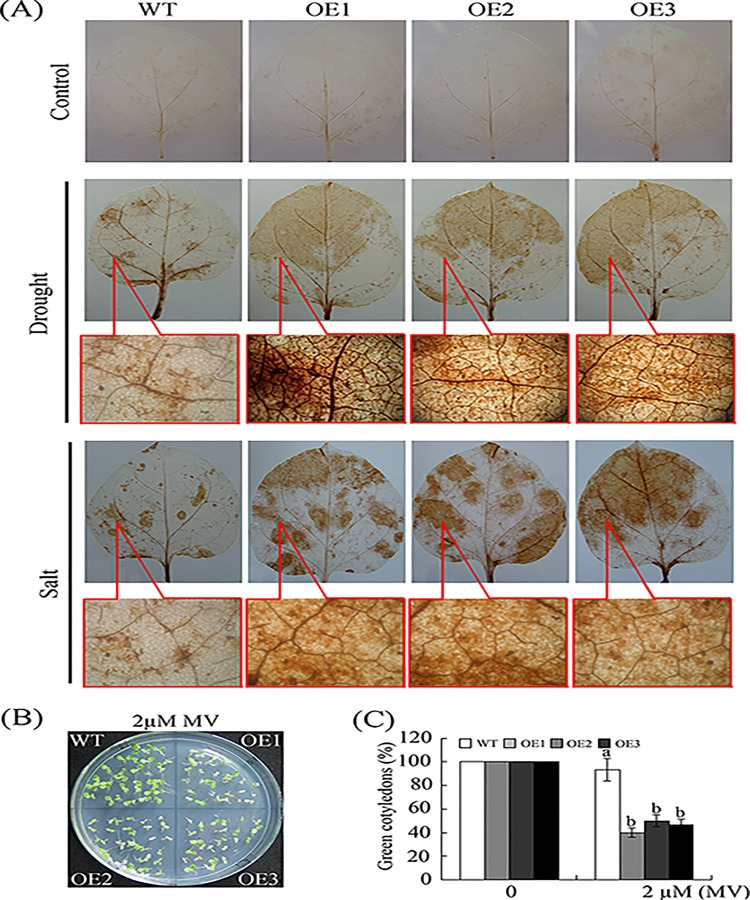
The constitutive expression of *GhWRKY68* increased ROS production and oxidative damage in transgenic plants. (A) Representative photographs of H_2_O_2_ accumulation using DAB staining. For drought and salinity treatments, the top figures show H_2_O_2_ accumulation, and the bottom figures illustrate the microscopic observations of the brown precipitate. (B) Phenotypes of young seedlings grown on medium containing 2 μM MV. (C) Quantification of cotyledon greening in the seedlings described in (B). The data represent the means ± SE of three independent experiments. The different letters above the columns in (C) indicate significant differences (*P* < 0.05) according to Duncan’s multiple range test performed using SAS version 9.1 software.

## Supporting information

S1 Fig Identification of transgenic plants. (A) Evaluation of transgenic plants in the T_0_ progeny of transgenic plants by RT-PCR. (B-C) Analysis of *GhWRKY68* expression in wild-type (WT) and T_1_ OE plants.
(TIF)

## References

[1] Jia H, Wang C, Wang F, Liu S, Li G, Guo X (2015) *GhWRKY68* Reduces Resistance to Salt and Drought in Transgenic *Nicotiana benthamiana*. PLoS ONE 10(3): e0120646 10.1371/journal.pone.0120646 25793865 PMC4368093

