# Supplementary material for: GhWRKY68 Reduces Resistance to Salt and Drought in Transgenic Nicotiana benthamiana
Source: PLoS One. 2015 Mar 20;10(3):e0120646. doi: 10.1371/journal.pone.0120646 (PMC4368093; doi:10.1371/journal.pone.0120646)
Supplement: S1 Table — (DOC) [file pone.0120646.s001.doc]

**Table S1.** Details of the primers used in this study

| **Abbreviation** | **Primer sequence (5′-3′)** | **Description** |
| --- | --- | --- |
| WP1 | GGGTTYATGGAGYTACTG | Degenerate primer, forward |
| WP2 | GGRTGDGTRTGTTGDCCTTC | Degenerate primer, reverse |
| 5W1 | CTCTCAACTCTTTTCTTCAC | 5′RACE reverse primer, primary |
| 5W2 | CTCGGTTCTCTCTGTCTC | 5′RACE reverse primer, nested |
| AAP | GGCCACGCGTCGACTAGTAC(G)_14_ | Abridged Anchor Primer |
| AUAP | GGCCACGCGTCGACTAGTAC | Abridged universal ampliﬁcation primer |
| 3W1 | GGTTACAGATGGAGAAAGTAC | 3′RACE forward primer, primary |
| 3W2 | CGTTGCACCACCATCTCATG | 3′RACE reverse primer, nested |
| B26 | GACTCTAGACGACATCGA(T)_18_ | Universal primer, primary |
| B25 | GACTCTAGACGACATCGA | Universal primer, nested |
| WQC1 | GATGAACAAGAACTGGAGT | Genomic sequence primer, forward |
| WQC2 | GCAGTAATCTCCACTTTGT | Genomic sequence primer, reverse |
| WG1 | TCTAGAATGGAGAGAAAACAGGGTGTA | Subcellular localization primer, forward |
| WG2 | CTCGAGCTCTTCTTTCAGCATATGGGA | Subcellular localization primer, reverse |
| WF1 | GTATTTTCCGTCTATGTGAGCC | I-PCR outer primer, forward |
| WF2 | GGTTATAGATGGAGAAA*GTAC*GG | I-PCR inner primer, forward |
| WR1 | CATCTTCCACCTTTACACCCTG | I-PCR outer primer, reverse |
| WR2 | CCATGTATCAATCAAACACTCC | I-PCR inner primer, reverse |
| WQ1 | CAGCACACTCATCCCAGTCC | Real-time Q- PCR primer, forward |
| WQ2 | AGAGGCGATTTTGCAGCC | Real-time Q-PCR primer, reverse |
| UBQ1 | CCAGAAGGAATCCACTTTGC | Cotton standard control primer, forward |
| UBQ2 | CCAGCTCACATCAGCATACG | Cotton standard control primer, reverse |
| WOE1 | TCTAGAATGGAGAGAAAACAGGGTGTA | Primers of constructing expression vector, forward |
| WOE2 | GAGCTCCTCTTCTTTCAGCATATGGGA | Primers of constructing expression vector, reverse |
| Nbactin F | TGGACTCTGGTGATGGTGTC |  |
| Nbactin R | CCTCCAATCCAAACACTGTA |  |
| NbAREB F | TTGCTGGTGGAAATGTAAGTGC |  |
| NbAREB R | GGAATGTAACATCCTTTGAGTATCG |  |
| NbDREB F | GAATAACCCCAAGAGGCG |  |
| NbDREB R | AGTCAGCGAAGTTCAAGCAA |  |
| NbAPX F  NbAPX R | GGAGTGGTTGCTGTTGAAGTC  GGAGAGCCTTGTCTGATGG |  |
| NbCAT F | cacagccacgctactcaagac |  |
| NbCAT R | CCACCCACCGACGAATAAAG |  |
| NbGST F | AGCACCCTTACCTTTCCCTC |  |
| NbGST R | GCAGCTTCTCCAATCCCTTAAC |  |
| NbSOD F | caactccacggcttccagac |  |
| NbSOD R | TGGGTCCTGATTAGCAGTGGT |  |
| NbRbohA F | ACACACGCCATCAGAACTCCA |  |
| NbRbohA R | CCCACCCAACCAAAATACGC |  |
| NbRbohB F | GTTTGCCAGCCACCACCTAAT |  |
| NbRbohB R | AAGAGCAGAACGAGCATCACC |  |
| NbNCED F | CGACCCACGAGTCCAGATTTC |  |
| NbNCED R | GAGCCTAGCAATTCCCGAGTG |  |
| Nbosmotin F | TTCAATGCTGCTGGTAGGGG |  |
| Nbosmotin R | GGTTAGTCGGGGCGAAAGTC |  |
| NbERD F | CACTGATAAGAACTATGCGTTCAC |  |
| NbERD R | CTAAGCTAATCACATTCAGCGAG |  |
| NbSnRK2.3F | GGCTAGGCTAGTTAAGGACAAGA |  |
| NbSnRK2.3R | GTTCTCCACCTGCTGCATACTCC |  |
| NbLEA F | GGATCTAATTGACAAGGCGAAG |  |
| NbLEA R | CTCGCCGCTATAAGAGAGAG |  |
