# Supplementary figures and images for: GhWRKY68 Reduces Resistance to Salt and Drought in Transgenic Nicotiana benthamiana
Source: PLoS One. 2015 Mar 20;10(3):e0120646. doi: 10.1371/journal.pone.0120646 (PMC4368093; doi:10.1371/journal.pone.0120646)

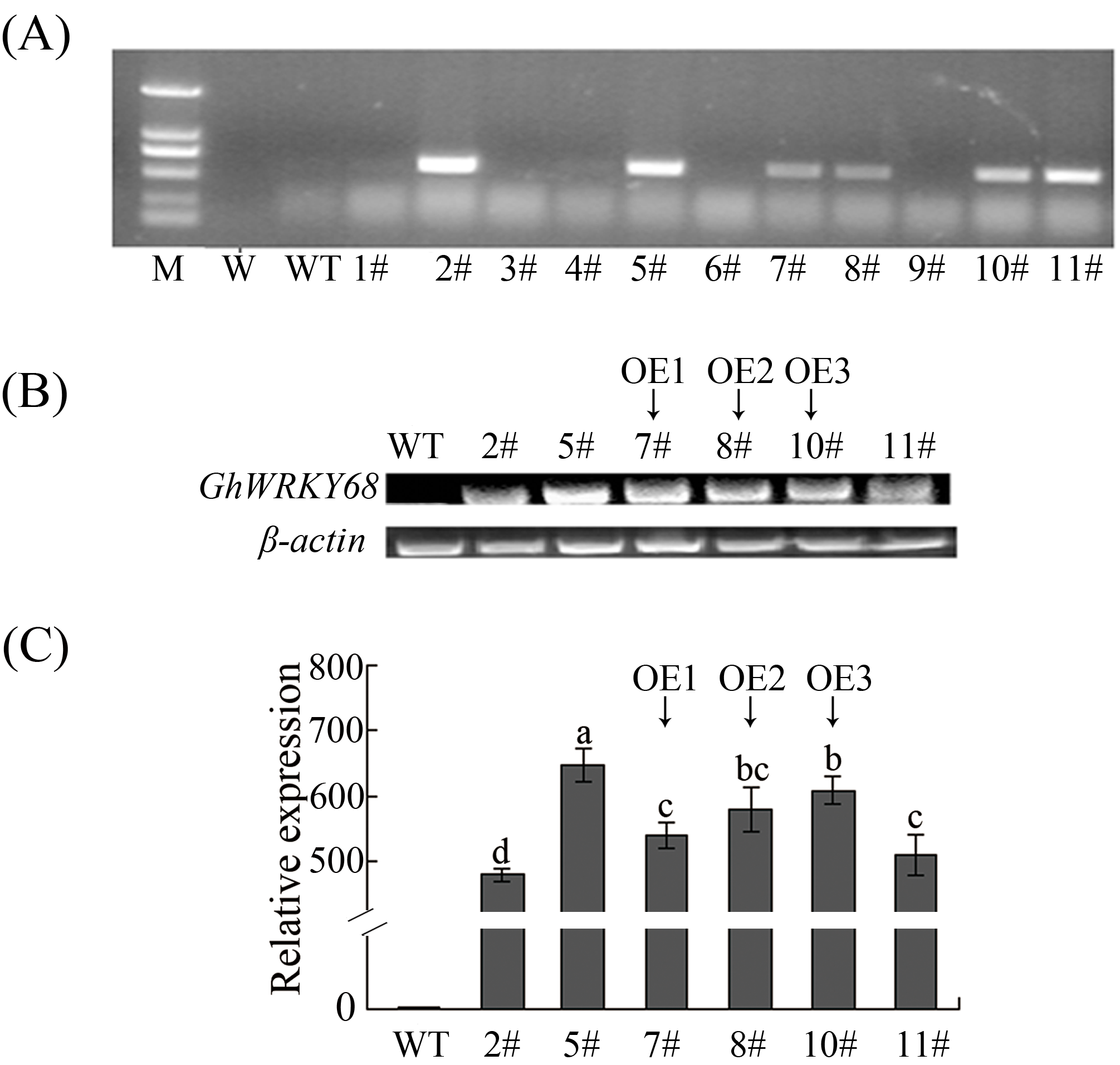

Supplement: S1 Fig — (A) Evaluation of transgenic plants in the T0 progeny of transgenic plants by RT-PCR. (B) Analysis of GhWRKY68 expression in wild-type (WT) and T1 OE plants. (TIF) [file pone.0120646.s002.tif]
