# Supplementary figures and images for: Correction: GhWRKY68 Reduces Resistance to Salt and Drought in Transgenic Nicotiana benthamiana
Source: PLoS One. 2019 Mar 12;14(3):e0213540. doi: 10.1371/journal.pone.0213540 (PMC6413924; doi:10.1371/journal.pone.0213540)

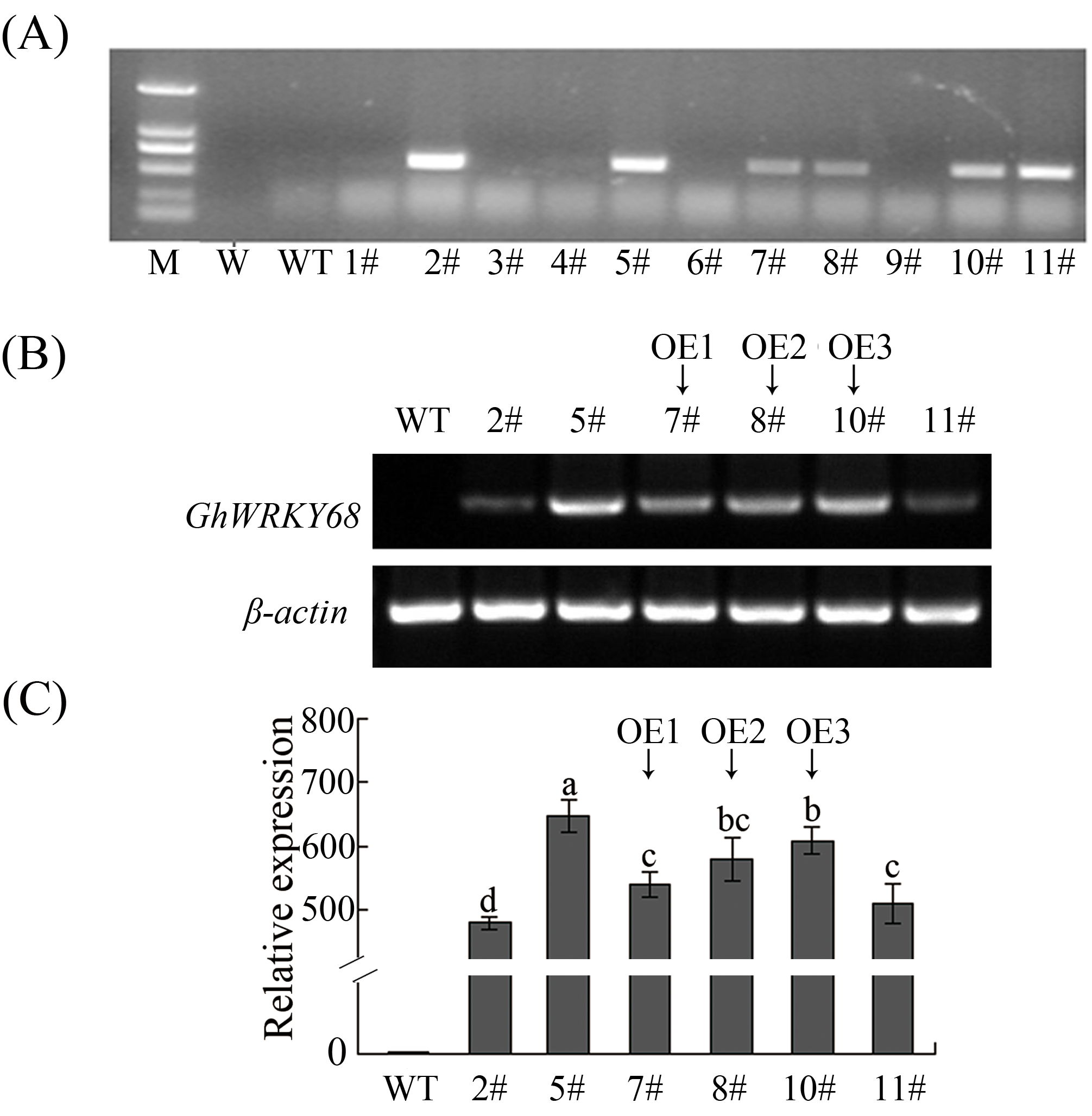

Supplement: S1 Fig — (A) Evaluation of transgenic plants in the T0 progeny of transgenic plants by RT-PCR. (B-C) Analysis of GhWRKY68 expression in wild-type (WT) and T1 OE plants. (TIF) [file pone.0213540.s001.tif]
